# Evaluation of Efficient and Practical Methods for the Preparation of Functionalized Aliphatic Trifluoromethyl Ethers

**DOI:** 10.3390/molecules22050804

**Published:** 2017-05-14

**Authors:** Taras M. Sokolenko, Maya I. Dronkina, Emmanuel Magnier, Lev M. Yagupolskii, Yurii L. Yagupolskii

**Affiliations:** 1 Institute of Organic Chemistry, NAS of Ukraine, Murmans’ka St. 5, Kiev 02660, Ukraine; taras_sk@ukr.net; 2 Bâtiment Lavoisier, Université de Versailles-Saint-Quentin, 45 Avenue des Etats-Unis, Versailles 78035, France; emmanuel.magnier@uvsq.fr; 3 Enamine Ltd, A. Matrosova str. 23, Kiev 01103, Ukraine

**Keywords:** fluorination, chlorination, oxidative desulfurization-fluorination, antimony trifluoride, hydrogen fluoride, trifluoromethyl ethers

## Abstract

The “chlorination/fluorination” technique for aliphatic trifluoromethyl ether synthesis was investigated and a range of products with various functional groups was prepared. The results were compared with oxidative desulfurization-fluorination of xanthates with the same structure.

## 1. Introduction

Fluorine is one of the most favorite heteroatoms for incorporation into small molecules in life science-oriented research. In particular, commercial pharmaceuticals and agrochemicals frequently contain single fluorine atoms or trifluoromethyl groups [1,2,3,4]. Nevertheless, growing interest in emergent fluorinated substituents, like α-fluorinated ethers, has been observed within recent years [5,6,7,8].

While aromatic trifluoromethyl ethers, since their first publication [9] in 1955, have been extensively studied and widely used as pharmaceuticals and crop protection agents, as well as critical components for liquid crystals design [3,4,5,6,10], aliphatic trifluoromethyl ethers are less studied. Only a few practical methods for the synthesis of such compounds have been developed. Fluorination of fluoroformates with SF_4_ leads to aliphatic trifluoromethyl ethers. For instance, methyl(trifluoromethoxy) acetate was obtained by this method [11]. Nucleophilic substitution of benzylic halogen or α-halogen atoms in acetophenones with perfluoroalcoholate anions is a suitable method for aliphatic ether preparation [12].

Oxidative desulfurization-fluorination of xanthates is now the most attractive method for primary alkyl trifluoromethyl ether synthesis. At least 42 publications concerning this reaction are cited in Reaxys. *N*-Bromo- [13] or *N*-iodosuccinimide [14], dibromodimethylhidantoine [15,16], and IF_5_ [17] are suitable oxidants in this reaction. Complexes pyridine-HF or NEt_3_-HF are commonly used as fluorine atom sources. Bromine trifluoride [18] or p-nitrophenylsulfur chlorotetrafluoride [19] were used as oxidants and fluorinating agents.

A number of new methods for trifluoromethyl ether synthesis were developed over the past ten years. The progress in this field was well summarized in the [6]. It is also worth noting the recent publication concern to asymmetric bromotrifluoromethoxylation of alkenes [8].

Surprisingly, and to the best of our knowledge, common for aromatic and revisited for heterocycles [20] trifluoromethyl ether synthesis “chlorination/fluorination” technique was not investigated enough for aliphatic substrates in order to compare existing methods due to the necessity to choose the most effective ones leading to the best yields and the lowest percentage of byproducts. It was reported that only 1,1,1-trifluoro-2-(trichloromethoxy)propane fluorination with anhydrous HF yielded the corresponding trifluoromethyl ether [21]. The aim of this work was to answer the question “is a “chlorination/fluorination” technique suitable for the aliphatic trifluoromethoxy-containing compound preparation as it is for aromatic ones” and to compare results of this method with oxidative desulfurization-fluorination at the same substrates.

## 2. Results and Discussion

We based our research on hydroxyacetic and β-hydroxypropionic acid derivatives, and alcohols containing two and three carbon atoms, including branched structures with phtalimido end groups as model objects.

**Scheme 1:**
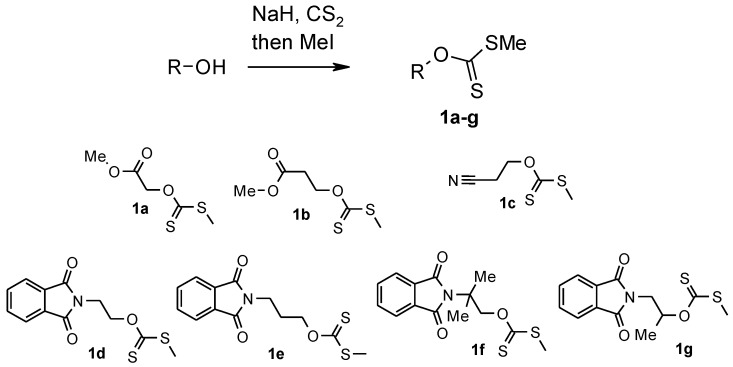
Synthesis and structures of methylxanthates.

Xanthates **1a**–**g** were prepared in a one-pot procedure starting from sodium alcoholates of ω-hydroxysubstituted aliphatic esters, nitriles, and N-protected alkanolamines by the action of carbon disulfide and methyliodide. Target products were obtained with high yields in all cases (Scheme 1).

**Scheme 2:**
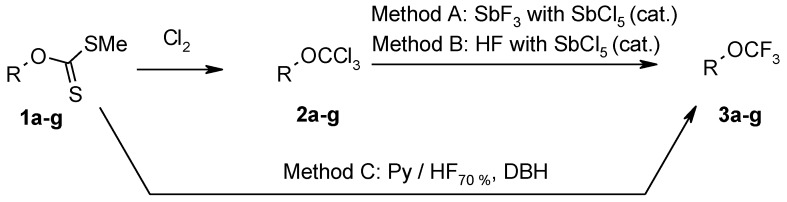
Syntheses of trifluoromethoxiderivatives.

Chlorination of xanthates **1a**–**g** readily occurred with elemental chlorine to produce trichloromethylethers **2a**–**g**, which were isolated with almost quantitative yields (Scheme 2). Chloroderivatives **2a**–**g** can be stored in a fridge and they are extremely sensitive to moisture.

Antimony trifluoride (method A) and hydrogen fluoride (method B) were used for chlorine-fluorine exchange because they are the cheapest for this reaction. Antimonium pentachloride was used as catalyst in both cases. Pyridine-HF_70%_ and dibromodimethylhidantoine (DBH) (method C) were used for oxidative desulfurization-fluorination. It must be added that xanthates **1a**–**g** were converted to trifluoromethyl ethers by the last method in the one-pot synthesis (Scheme 2).

In the case of trichloromethoxyacetate **2a** fluorination by both methods A and B failed. The desired compound **3a** was prepared with low yield only by oxidative desulfurization-fluorination of xanthate **1a** (Table 1, Entry 1). On the contrary, fluorination of trichloromethoxypropanecarboxylic acids derivatives **2b**–**c** and xanthates **1b**–**c** was successfully performed by all three methods (Table 1, entry 2,3). In the case of nitrile **2c** reaction with SbF_3_ (method A) lead to low yield of trifluoromethylether **3c** (Table 1, Entry 4). Thus, the presence of electron-withdrawing carboxylic group close to the reaction center prevented successful fluorination processes.

In general, fluorination of trichloromethylethers **2d**–**g** and xanthates **1d**–**g** with protected amino groups (Table 1, Entry 4–7) results in higher yields of fluorinated products **3d**–**g** as compared with the same reaction of carboxylic acids derivatives. Trifluoromethylethers with linear chains **3d,e** were prepared with high yields by all three methods. Oxidative desulfurization-fluorination of xanthate **1f** gave a better result than fluorination of chloroderivative **2f** with SbF_3_ or HF (Table 1, entry 6). An essential difference was observed in fluorination of trichloromethylether **2g** and xanthate **1g** (Table 1, entry 7). Fluorination of compound **2g** by methods A and B led to low yields of trifluoromethylether **3g**. Nevertheless, oxidative desulfurization-fluorination of xanthate **1g** allowed obtaining ether **3g** with quantitative yield. It should be noted that both substrates (**1g** and **2g**) are secondary alcohol derivatives.

It must be added that fluorination with pyridine-HF complex (method C) requires special polyethylene or polytetrafluoroethylene plastic equipment, fluorination with anhydrous HF (method B) require stainless steel autoclave but fluorination with antimony trifluoride (method A) was performed in common glass equipment. All investigated methods of fluorination (A, B, and C) can be applied for the large-scale experiment. Thus, methyl 3-(trifluoromethoxy)propanoate **3b** was prepared in quantity above 25 g at once and protected amine **3d** was obtained in ca. 50 g at once by all three methods. The protective phthalimide group can be removed by common methods and was documented in [16].

## 3. Materials and Methods

^1^H-NMR spectra were recorded at 300 MHz with a Varian VXR-300 spectrometer (Varian Inc, Palo Alto, CA, USA), at 500 MHz with a Bruker AVANCE DRX 500 instrument (Bruker, Billerica, MA, USA), or at 400 MHz with Varian UNITY-Plus 400 spectrometer (Varian Inc, Palo Alto, CA, USA). ^13^C-NMR‑spectra (proton decoupled) were recorded on a Bruker AVANCE DRX 500 instrument at 125 MHz, or at 100 MHz with Varian UNITY-Plus 400 spectrometer. ^19^F-NMR spectra were recorded at 188 MHz with a Varian Geminy-200 instrument (Varian Inc., Palo Alto, CA, USA), or at 376 MHz with Varian UNITY-Plus 400 spectrometer. Chemical shifts are given in ppm relative to Me_4_Si and CCl_3_F, respectively, as internal or external standards. ^1^H-, ^13^C-, and ^19^F-NMR spectra of the compounds can be found in Supplementary materials. LC-MS spectra were registered on an “Agilent 1100 Series” instrument with diode-matrix and mass-selective detector “Agilent 1100 LS/MSD SL” (ionization method: chemical ionization at atmospheric pressure; ionization chamber operation conditions: simultaneous scanning of positive and negative ions in the range 80–1000 *m*/*z*, Agilent Technologies, Santa Clara, CA, USA). GC-MS spectra were registered on a Hewlett-Packard HP GC/MS 5890/5972 instrument (EI 70 eV) (Philips, Bothell, WA, USA). Melting points were determined in open capillaries using an SMP3 instrument (Stuart Scientific Bibby Sterlin Ltd, Stone, Staffordshire, UK). Elemental analysis was performed in the Analytical Laboratory of the Institute of Organic Chemistry, NAS of Ukraine, Kiev.

Unless otherwise stated, commercially-available reagents were purchased from Enamine Ltd. and were used without purification. Antimony trifluoride was sublimated immediately prior to use. Anhydrous hydrogen fluoride was distilled in the presences of SOCl_2_. HF_70%_/pyridine was prepared according to the Olah method [22]. Solvents were dried before use by standard methods. All reactions were performed in an argon atmosphere. For column chromatography, Merck Kieselgel 60 silica gel (Merck, Darmstadt, Germany) was used. Thin-layer chromatography (TLC) was carried out on aluminum-backed plates coated with silica gel (Merck Kieselgel 60 F254, Merck, Darmstadt, Germany).

### 3.1. Synthesis of Xanthates ***1a–c***: General Procedure

Carbon disulfide (18.1 mL, 0.3 mol) was added dropwise at −30 °C to the suspension of sodium hydride (60% in mineral oil) (4.4 g, 0.11 mol) in anhydrous dimethylformamide (DMF) (200 mL). To this mixture the solution of corresponding hydroxyderivatives (0.1 mol) in anhydrous DMF (15 mL) was added dropwise for 3 h with mechanistic stirring at −30 °C. After addition was completed, the mixture was warmed to room temperature and stirred for 3 h. The color of the mixture gradually changed to dark red. The reaction mixture was cooled to −10 °C and methyl iodide (7.8 mL, 0.12 mol) was added dropwise. The mixture was warmed to room temperature, stirred for 3 h until the color changed from dark red to yellow and poured into ice (600 g), and extracted with tert-buthylmethyl ether (MTBE) (5 × 50 mL). The extract was washed with brine (5 × 50 mL) and dried with MgSO_4_. The solvent was removed at atmospheric pressure and the residue distilled in vacuum.

#### 3.1.1. Methyl ([(methylsulfanyl)carbonothioyl]oxy)acetate **1a**

Yield 15.5 g (86%). Yellow oil: bp 129–130 °C (20 Torr). ^1^H-NMR (400 MHz, CDCl_3_): δ 2.60 (s, 3H, SCH_3_), 3.77 (s, 3H, OCH_3_), 5.15 (s, 2H, CH_2_). ^13^C-NMR (100 MHz, CDCl_3_): δ 19.5 (s, SCH_3_), 52.4 (s, OCH_3_), 67.6 (s, CH_2_), 167.1 (s, C=O), 215.8 (s, C=S). GC-MS, 70 eV, *m*/*z* (rel. int.): 180 (69) [M]^+^. Anal. calcd for C_5_H_8_O_3_S_2_: C, 33.32; H, 4.47; S, 35.58; found: C, 33.14; H, 4.51; S, 35.51.

#### 3.1.2. Methyl 3-([(methylsulfanyl)carbonothioyl]oxy)propanoate **1b**

Yield 17.8 g (92%). Yellow oil: bp 94–95 °C (0.5 Torr). ^1^H-NMR (400 MHz, CDCl_3_): δ 2.52 (s, 3H, SCH_3_), 2.80 (t, ^3^*J* = 6.4 Hz, 2H, CH_2_), 3.70 (s, 3H, OCH_3_), 5.15 (t, ^3^*J* = 6.4 Hz, 2H, CH_2_). ^13^C-NMR (125 MHz, CDCl_3_): δ 18.7 (s, SCH_3_), 32.9 (s, CH_2_), 51.6 (s, OCH_3_), 68.2 (s, CH_2_), 170.2 (s, C=O), 215.2 (s, C=S). GC-MS, 70 eV, *m*/*z* (rel. int.): 194 (62) [M]^+^. Anal. calcd for C_6_H_10_O_3_S_2_: C, 37.09; H, 5.19; S, 33.01; found: C, 37.17; H, 5.29; S, 32.94.

#### 3.1.3. *O*-(2-Cyanoethyl)-*S*-methyl-dithiocarbonate **1c**

Yield 14.3 g (89%). Yellow oil: bp 94–95 °C (0.3 Torr). ^1^H-NMR (300 MHz, CDCl_3_): δ 2.53 (s, 3H, SCH_3_), 2.82 (t, ^3^*J* = 6.3 Hz, 2H, CH_2_), 4.73 (t, ^3^*J* = 6.3 Hz, 2H, CH_2_). ^13^C-NMR (125 MHz, CDCl_3_): δ 17.3 (s, CH_2_), 18.9 (s, SCH_3_), 66.2 (s, CH_2_), 116.1 (s, C≡N), 214.9 (s, C=S). GC-MS, 70 eV, *m*/*z* (rel. int.): 161 (67) [M]^+^. Anal. calcd for C_5_H_7_NOS_2_: C, 37.24; H, 4.38; S, 39.77; found: C, 37.30; H, 5.44; S, 39.70.

### 3.2. Synthesis of Xanthates ***1d**–**g***: General Procedure

To the stirred solution of hydroxyderivatives (0.1 mol) in anhydrous DMF (50 mL) sodium hydride (60% in mineral oil) (4.4 g, 0.11 mol) was added with mechanistic stirring between −5 and 0 °C. After addition, stirring was continued for 2 h at room temperature. Carbon disulfide (9 mL, 0.15 mol) was added dropwise at 0 °C. The reaction mixture was warmed to room temperature and stirred for 5 h. The color of the one was gradually changed to dark red. The reaction mixture was cooled to 0 °C and methyl iodide (7.8 mL, 0.12 mol) was added dropwise. The mixture was warmed to room temperature, stirred for 3 h until the color changed from dark red to yellow and poured into ice (100 g), extracted with CH_2_Cl_2_ (3 × 50 mL). The extract was washed with water (3 × 50 mL) and dried with MgSO_4_. After removal of the solvent the product was washed with hexane and dried in vacuum.

#### 3.2.1. *O*-[2-(1,3-Dioxo-1,3-dihydro-2*H*-isoindol-2-yl)ethyl] *S*-Methyl Dithiocarbonate **1d**

Yield 27.8 g (99%). Yellow powder: mp 105–106 °C. ^1^H-NMR (400 MHz, CDCl_3_): δ 2.49 (s, 3H, SCH_3_), 4.10 (t, ^3^*J* = 5.6 Hz, 2H, CH_2_), 4.82 (t, ^3^*J* = 5.6 Hz, 2H, CH_2_), 7.71–7.73 (m, 2H, arom H), 7.83–7.86 (m, 2H, arom H). ^13^C-NMR (125 MHz, DMSO-d_6_): δ 18.4 (s, SCH_3_), 36.3 (s, CH_2_), 70.6 (s, CH_2_), 123.2 (s, arom. C), 131.6 (s, arom. C), 134.6 (s, arom. C), 167.7 (s, C=O), 215.2 (s, C=S). LC-MS, *m*/*z*: 282 [M + H]^+^. Anal. calcd for C_12_H_11_NO_3_S_2_: C, 51.23; H, 3.94; N, 4.98; S, 22.79; found: C, 51.30; H, 4.00; N, 4.98; S, 22.70.

#### 3.2.2. *O*-[3-(1,3-Dioxo-1,3-dihydro-2*H*-isoindol-2-yl)propyl] *S*-Methyl Dithiocarbonate **1e**

Yield 29.5 g (100%). Yellow powder: mp 101–102 °C. ^1^H-NMR (300 MHz, DMSO-d_6_): δ 2.08–2.12 (m, 2H, CH_2_), 3.34 (s, 3H, SCH_3_), 3.71 (t, ^3^*J* = 6.3 Hz, 2H, CH_2_), 4.58 (t, ^3^*J* = 6.0 Hz, 2H, CH_2_), 7.83–7.85 (m, 4H, arom H). ^13^C-NMR (100 MHz, DMSO-d_6_): δ 18.8 (s, SCH_3_), 27.3 (s, CH_2_), 35.0 (s, CH_2_), 72.4 (s, CH_2_), 123.4 (s, arom. C), 132.1 (s, arom. C), 134.7 (s, arom. C), 168.3 (s, C=O), 215.6 (s, C=S). LC-MS, *m*/*z*: 296 [M + H]^+^. Anal. calcd for C_13_H_13_NO_3_S_2_: C, 52.86; H, 4.44; N, 4.74; S, 21.71; found: C, 52.92; H, 4.52; N, 4.68; S, 21.70.

#### 3.2.3. *O*-[2-(1,3-Dioxo-1,3-dihydro-2*H*-isoindol-2-yl)-2-methylpropyl] *S*-Methyl Dithiocarbonate **1f**

Yield 27.5 g (89%). Yellow powder: mp 85–86 °C. ^1^H-NMR (300 MHz, CDCl_3_): δ 1.77 (s, 6H, 2CH_2_), 2.47 (s, 3H, SCH_3_), 4.98 (s, 2H, CH_2_), 7.70–7.73 (m, 2H, arom H), 7.78–7.81 (m, 2H, arom H). ^13^C-NMR (125 MHz, CDCl_3_): δ 18.5 (s, SCH_3_), 26.2 (s, 2CH_3_), 58.7 (s, C), 77.1 (s, CH_2_), 122.5 (s, arom. C), 131.4 (s, arom. C), 133.6 (s, arom. C), 168.8 (s, C=O), 214.7 (s, C=S). LC-MS, *m*/*z*: 310 [M + H]^+^. Anal. calcd for C_14_H_15_NO_3_S_2_: C, 54.35; H, 4.89; N, 4.53; S, 20.73; found: C, 54.42; H, 4.96; N, 4.50; S, 20.75.

#### 3.2.4. *O*-[2-(1,3-Dioxo-1,3-dihydro-2*H*-isoindol-2-yl)-1-methylethyl] *S*-Methyl Dithiocarbonate **1g**

Yield 28.9 g (98%). Yellow powder: mp 85–86 °C. ^1^H-NMR (300 MHz, CDCl_3_): δ 1.43 (d, ^3^*J* = 6.4 Hz, 3H, CH_3_), 2.48 (s, 3H, SCH_3_), 3.89 (dd, ^2^*J* = 14.4 Hz, ^3^*J* = 3.2 Hz, 1H, CH_2_), 4.04 (dd, ^2^*J* = 14.4 Hz, ^3^*J* = 7.6 Hz, 1H, CH_2_), 6.00–7.10 (m, 1H, CH), 7.70–7.72 (m, 2H, arom H), 7.78–7.81 (m, 2H, arom H). ^13^C-NMR (125 MHz, CDCl_3_): δ 17.5 (s, CH_3_), 18.9 (s, SCH_3_), 43.7 (s, CH_2_), 77.6 (s, C), 123.5 (s, arom. C), 132.0 (s, arom. C), 134.2 (s, arom. C), 168.1 (s, C=O), 215.3 (s, C=S). LC-MS, *m*/*z*: 296 [M + H]^+^. Anal. calcd for C_13_H_13_NO_3_S_2_: C, 52.86; H, 4.44; N, 4.74; S, 21.71; found: C, 52.88; H, 4.86; N, 4.69; S, 21.76.

### 3.3. Synthesis of Trichloroderivatives ***2a–g***: General Procedure

Chlorine was bubbled through the solution of xanthate **1a**–**g** (50 mmol) in carbon tetrachloride (50 mL) at 0 °C for 1 h. The reaction mixture was warmed to 20 °C and chlorine was bubbled for further 1 h. Methylene chloride (10 mL) was added to the reaction mixture and chlorine was bubbled for further 1 h. Excess of chlorine was removed with stream of nitrogen. After removal of the solvent, product was distilled in vacuum or washed with hexane and dried in vacuum.

#### 3.3.1. Methyl (trichloromethoxy)acetate **2a**

Yield 9.0 g (87%). Colorless liquid: bp 87–88 °C (10 Torr). ^1^H-NMR (400 MHz, CDCl_3_): δ 3.83 (s, 3H, OCH_3_), 4.64 (s, 2H, CH_2_). ^13^C-NMR (125 MHz, CDCl_3_): δ 52.7 (s, OCH_3_), 66.7 (s, CH_2_), 112.6 (s, CCl_3_), 166.2 (s, C=O). Anal. calcd for C_4_H_5_Cl_3_O_3_: C, 23.16; H, 2.43; Cl, 51.27; found: C, 33.12; H, 2.40; Cl, 52.35.

#### 3.3.2. Methyl 3-(trichloromethoxy)propanoate **2b**

Yield 9.8 g (89%). Colorless liquid: bp 64–65 °C (0.5 Torr). ^1^H-NMR (300 MHz, CDCl_3_): δ 2.76 (t, ^3^*J* = 6.4 Hz, 2H, CH_2_), 3.71 (s, 3H, OCH_3_), 5.35 (t, ^3^*J* = 6.4 Hz, 2H, CH_2_). ^13^C-NMR (125 MHz, CDCl_3_): δ 32.9 (s, CH_2_), 51.7 (s, OCH_3_), 66.4 (s, CH_2_), 111.9 (s, CCl_3_), 169.7 (s, C=O). Anal. calcd for C_5_H_7_Cl_3_O_3_: C, 27.12; H, 3.19; Cl, 48.02; found: C, 27.07; H, 3.12; Cl, 48.09.

#### 3.3.3. 3-(Trichloromethoxy)propanenitrile **2c**

Yield 8.7 g (92%). Colorless liquid: bp 77–78 °C (0.3 Torr). ^1^H-NMR (300 MHz, CDCl_3_): δ 2.83 (t, ^3^*J* = 6.6 Hz, 2H, CH_2_), 4.29 (t, ^3^*J* = 6.6 Hz, 2H, CH_2_). ^13^C-NMR (125 MHz, CDCl_3_): δ 17.6 (s, CH_2_), 64.8 (s, CH_2_), 111.9 (s, CCl_3_), 115.5 (s, C≡N). Anal. calcd for C_4_H_4_Cl_3_NO: C, 25.50; H, 2.14; Cl, 56.44; N, 7.43; found: C, 25.55; H, 2.14; Cl, 56.50; N, 7.40.

#### 3.3.4. 2-[2-(Trichloromethoxy)ethyl]-1*H*-isoindole-1,3(2*H*)-dione **2d**

Yield 14.8 g (96%). Colorless powder: mp 93–94 °C. ^1^H-NMR (300 MHz, CDCl_3_): δ 4.06 (t, ^3^*J* = 5.3 Hz, 2H, CH_2_), 4.34 (t, ^3^*J* = 5.3 Hz, 2H, CH_2_), 7.74–7.76 (m, 2H, arom H), 7.83–7.86 (m, 2H, arom H). ^13^C-NMR (100 MHz, CDCl_3_): δ 36.4 (s, CH_2_), 68.1 (s, CH_2_), 112.5 (s, CCl_3_), 123.5 (s, arom. C), 131.8 (s, arom. C), 134.2 (s, arom. C), 167.8 (s, C=O). Anal. calcd for C_11_H_8_Cl_3_NO_3_: C, 42.82; H, 2.61; Cl, 34.47; N, 4.54; found: C, 42.90; H, 2.52; Cl, 34.50; N, 4.44.

#### 3.3.5. 2-[3-(Trichloromethoxy)propyl]-1*H*-isoindole-1,3(2*H*)-dione **2e**

Yield 15.3 g (95%). Colorless powder: mp 102–103 °C. ^1^H-NMR (400 MHz, CDCl_3_): δ 2.12–2.19 (m, 2H, CH_2_), 3.83 (t, ^3^*J* = 6.8 Hz, 2H, CH_2_), 4.16 (t, ^3^*J* = 6.0 Hz, 2H, CH_2_), 7.60–7.70 (m, 2H, arom H), 7.80–7.90 (m, 2H, arom H). ^13^C-NMR (125 MHz, CDCl_3_): δ 27.3 (s, CH_2_), 35.1 (s, CH_2_), 69.6 (s, CH_2_), 112.4 (s, CCl_3_), 123.3 (s, arom. C), 132.0 (s, arom. C), 134.0 (s, arom. C), 168.2 (s, C=O). Anal. calcd for C_12_H_10_Cl_3_NO_3_: C, 44.68; H, 3.12; Cl, 32.97; N, 4.34; found: C, 44.75; H, 3.10; Cl, 33.00; N, 4.40.

#### 3.3.6. 2-[1,1-Dimethyl-2-(trichloromethoxy)ethyl]-1*H*-isoindole-1,3(2*H*)-dione **2f**

Yield 13.8 g (82%). Colorless powder: mp 93–94 °C. ^1^H-NMR (500 MHz, CDCl_3_): δ 1.79 (s, 6H, 2CH_3_), 4.53 (s, 2H, CH_2_), 7.68–7.71 (m, 2H, arom H), 7.75–7.80 (m, 2H, arom H). ^13^C-NMR (125 MHz, CDCl_3_): δ 24.7 (s, 2CH_3_), 58.8 (s, C), 75.6 (s, CH_2_), 112.6 (s, CCl_3_), 123.1 (s, arom. C), 131.9 (s, arom. C), 134.1 (s, arom. C), 169.4 (s, C=O). Anal. calcd for C_13_H_12_Cl_3_NO_3_: C, 46.39; H, 3.59; Cl, 31.60; N, 4.16; found: C, 46.45; H, 3.50; Cl, 31.68; N, 4.05.

#### 3.3.7. 2-[2-(Trichloromethoxy)propyl]-1*H*-isoindole-1,3(2*H*)-dione **2g**

Yield 13.7 g (85%). Colorless powder: mp 96–97 °C. ^1^H-NMR (300 MHz, CDCl_3_): δ 1.55 (d, ^3^*J* = 6.3 Hz, 3H, CH_3_), 3.79 (dd, ^2^*J* = 14.1 Hz, ^3^*J* = 3.6 Hz, 1H, CH_2_), 4.06 (dd, ^2^*J* = 14.1 Hz, ^3^*J* = 8.1 Hz, 1H, CH_2_), 4.80–4.95 (m, 1H, CH), 7.70–7.80 (m, 2H, arom H), 7.85–7.95 (m, 2H, arom H). ^13^C-NMR (125 MHz, CDCl_3_): δ 18.4 (s, CH_3_), 42.1 (s, CH_2_), 78.2 (s, C), 123.5 (s, arom. C), 132.5 (s, arom. C), 134.2 (s, arom. C), 167.9 (s, C=O). Anal. calcd for C_12_H_10_Cl_3_NO_3_: C, 44.68; H, 3.12; Cl, 32.97; N, 4.34; found: C, 44.75; H, 3.20; Cl, 33.00; N, 4.35.

### 3.4. Fluorination of Trichloromethoxy Derivatives ***2a–g*** with Antimony Trifluoride (Method A)

#### 3.4.1. Fluorination of Esters **2a**–**b** and Nitrile **2c**: General Procedure

The mixture of pounded antimony trifluoride (17.9 g, 100 mmol) and antimonium pentachloride (1.5 g, 5 mmol) was carefully heated to 30−35 °C with mechanistic stirring to a homogenous pasty mass formation. The mixture was cooled to 0 °C and trichloromethoxy derivative **2a**–**c** (50 mmol) was added at once. The reaction mixture was heated at 90 °C for 10 min. Then the mixture was heated to 120−135 °C with simultaneous vacuum (150 Torr) distillation of the product in the trap cooled with crushed ice. The pure product was obtained after redistillation.

#### 3.4.2. Fluorination of Protected Amines **2d**–**g**: General Procedure

The mixture of pounded antimony trifluoride (17.9 g, 100 mmol) and antimonium pentacloride (1.5 g, 5 mmol) was carefully heated to 30−35 °C with mechanistic stirring to a homogenous pasty mass formation. The mixture was cooled to 0 °C and trichloromethoxy derivative **2d**–**g** (50 mmol) was added at once. The reaction mixture was gradually heated firstly at 25 °C for 30 min, then at 45 °C for 30 min, and finally at 95 °C for 20 min. After cooling to room temperature the reaction mixture was washed with CH_2_Cl_2_ (5 × 50 mL), combined organic extracts were washed with 10% aqueous HCl solution (10 × 20 mL) then with water (5 × 20 mL) and dried with MgSO_4_. The solution was filtered through SiO_2_ and evaporated to dryness. The pure product was obtained after crystallization from hexane.

### 3.5. Fluorination of Trichloromethoxy Derivatives ***2a–g*** with Hydrogenfluoride (Method B): General Procedure

Trichloromethoxy derivative **2a**–**g** (50 mmol) was placed into stainless steel autoclave (100 mL volume) and cooled to −40 °C. Anhydrous HF (50 mL) and SbCl_5_ (0.75 g, 2.5 mmol) were added at the same temperature. Autoclave was sealed and the reaction mixture was heated at 45 °C for 0.5 h, then at 95 °C for 2.5 h, and cooled to room temperature. Excess of hydrogen fluoride and hydrogen chloride formed in the reaction were distilled in a polyethylene trap cooled to −15 °C. The residue was dissolved in CH_2_Cl_2_ (100 mL) washed with saturated aqueous K_2_CO_3_ solution (4 × 20 mL), then with water (3 × 20 mL), dried with MgSO_4_, and filtered through SiO_2_. After removal of the solvent, the product was distilled in vacuum or crystallized from hexane and dried in vacuum.

### 3.6. Fluorination of Xanthate ***1a–g*** with Pyridine-Hydrogenfluoride and 1,3-Dibromo-5,5-Dimethylhydantoin (DBH) (Method C): General Procedure

Reactions were performed in a polyethylene flask (500 mL) equipped with an additional polyethylene funnel, magnetic stirrer, and BOLA PTFE coated temperature probe.

Complex HF_70%_-pyridine was added dropwise at −78 °C to a suspension of DBH (42.9 g, 0.15 mol) in CH_2_Cl_2_ (130 mL). The mixture was stirred for 10 min and the solution of corresponding xanthate **1a–g** (0.05 mol) in CH_2_Cl_2_ (70 mL) was added at the same temperature. The reaction mixture was stirred at −78 °C for 1 h, warmed to room temperature, stirred for 5 h and poured into ice (100 g). As saturated aqueous solution of Na_2_SO_3_ was added to the mixture until the red color of the mixture changed to light yellow. Then the mixture was neutralized with aqueous K_2_CO_3_ solution. The organic layer was separated and the aqueous solution was extracted with CH_2_Cl_2_ (3 × 50 mL). Combined organic extracts were washed with water (3 × 500 mL) and dried with MgSO_4_. After removal of the solvent, the product was distilled or crystallized from hexane and dried in vacuum.

#### 3.6.1. Methyl (trifluoromethoxy)acetate **3a**

Yield 2.84 g (36% method C). Colorless liquid: bp 102–104 °C (lit. 110 °C [11]). ^1^H-NMR (400 MHz, CDCl_3_): δ 3.81 (s, 3H, OCH_3_), 4.48 (s, 2H, CH_2_). ^13^C-NMR (125 MHz, CDCl_3_): δ 52.5 (s, OCH_3_), 62.9 (q, ^3^*J*_CF_ = 2.5 Hz, CH_2_), 121.4 (q, *J*_CF_ = 256.4 Hz, CF_3_), 166.5 (s, C=O). ^19^F-NMR (376.5 MHz, CDCl_3_): δ −62.12 (s, CF_3_). GC-MS, 70 eV, *m*/*z* (rel. int.): 158 (46) [M]^+^. Anal. calcd for C_4_H_5_F_3_O_3_: C, 30.39; H, 3.19; found: C, 30.12; H, 3.04.

#### 3.6.2. Methyl 3-(trifluoromethoxy)propanoate **3b**

Yield 4.55 g (53% method A). Yield 5.9 g (69% method B). Yield 5.4 g (63% method B). Colorless liquid: bp 124–125 °C. ^1^H-NMR (300 MHz, CDCl_3_): δ 2.64 (t, ^3^*J* = 5.4 Hz, 2H, CH_2_), 3.66 (s, 3H, OCH_3_), 4.17 (t, ^3^*J* = 5.4 Hz, 2H, CH_2_). ^13^C-NMR (125 MHz, CDCl_3_): δ 33.2 (s, CH_2_), 51.3 (s, OCH_3_), 62.2 (q, ^3^*J*_CF_ = 3.3 Hz, CH_2_), 121.0 (q, *J*_CF_ = 253.3 Hz, CF_3_), 169.8 (s, C=O). ^19^F-NMR (188 MHz, CDCl_3_): δ −61.88 (s, CF_3_). GC-MS, 70 eV, *m*/*z* (rel. int.): 172 (52) [M]^+^. Anal. calcd for C_5_H_7_F_3_O_3_: C, 34.89; H, 4.10; found: C, 34.55; H, 4.02.

#### 3.6.3. 3-(Trifluoromethoxy)propanenitrile **3c**

Yield 1.5 g (22% method A). Yield 4.6 g (66% method B). Yield 4.6 g (66% method B). Colorless liquid: bp 100–102 °C (120 Torr). ^1^H-NMR (300 MHz, CDCl_3_): δ 2.70 (t, ^3^*J* = 6.3 Hz, 2H, CH_2_), 4.11 (t, ^3^*J* = 6.3 Hz, 2H, CH_2_). ^13^C-NMR (125 MHz, CDCl_3_): δ 18.0 (s, CH_2_), 61.2 (q, ^3^*J*_CF_ = 3.4 Hz, CH_2_), 115.3 (s, C≡N), 120.8 (q, *J*_CF_ = 255.1 Hz, CF_3_). ^19^F-NMR (188 MHz, CDCl_3_): δ −62.10 (s, CF_3_). GC-MS, 70 eV, *m*/*z* (rel. int.): 139 (66) [M]^+^. Anal. calcd for C_4_H_4_F_3_NO: C, 34.54; H, 2.90; N, 10.07; found: C, 34.40; H, 2.84; N, 9.90.

#### 3.6.4. 2-[2-(Trifluoromethoxy)ethyl]-1*H*-isoindole-1,3(2*H*)-dione **3d**

Yield 11.65 g (90% method A). Yield 12.0 g (93% method B). Yield 12.4 g (96% method B). Colorless powder: mp 73–74 °C (lit. 77 °C [16]), bp 100–102 °C (0.2 Torr). ^1^H-NMR (300 MHz, CDCl_3_): δ 3.93 (t, ^3^*J* = 5.3 Hz, 2H, CH_2_), 4.20 (t, ^3^*J* = 5.3 Hz, 2H, CH_2_), 7.70–7.77 (m, 2H, arom H), 7.80–7.90 (m, 2H, arom H). ^13^C-NMR (125 MHz, CDCl_3_): δ 36.7 (s, CH_2_), 63.9 (s, CH_2_), 121.7 (q, *J*_CF_ = 255.2 Hz, CF_3_), 123.5 (s, arom. C), 131.8 (s, arom. C), 134.2 (s, arom. C), 167.8 (s, C=O). ^19^F-NMR (188 MHz, CDCl_3_): δ −61.55 (s, CF_3_). LC-MS, *m*/*z*: 260 [M + H]^+^. Anal. calcd for C_11_H_8_F_3_NO_3_: C, 50.98; H, 3.11; N, 5.40; found: C, 50.90; H, 3.23; N, 5.44.

#### 3.6.5. 2-[3-(Trifluoromethoxy)propyl]-1*H*-isoindole-1,3(2*H*)-dione **3e**

Yield 12.0 g (88% method A). Yield 12.3 g (90% method B). Yield 13.0 g (96% method B). Colorless powder: mp 77–78 °C. ^1^H-NMR (400 MHz, CDCl_3_): δ 2.04–2.12 (m, 2H, CH_2_), 3.70–3.82 (m, 2H, CH_2_), 4.00–4.12 (m, 2H, CH_2_), 7.68–7.72 (m, 2H, arom H), 7.80–7.88 (m, 2H, arom H). ^13^C-NMR (125 MHz, CDCl_3_): δ 28.0 (s, CH_2_), 34.8 (s, CH_2_), 65.1 (s, CH_2_), 121.6 (q, *J*_CF_ = 254.0 Hz, CF_3_), 123.4 (s, arom. C), 132.1 (s, arom. C), 134.2 (s, arom. C), 168.3 (s, C=O). ^19^F-NMR (376.5 MHz, CDCl_3_): δ −61.49 (s, CF_3_). LC-MS, *m*/*z*: 274 [M + H]^+^. Anal. calcd for C_12_H_10_F_3_NO_3_: C, 52.75; H, 3.69; N, 5.13; found: C, 52.77; H, 3.70; N, 5.22.

#### 3.6.6. 2-[1,1-Dimethyl-2-(trifluoromethoxy)ethyl]-1*H*-isoindole-1,3(2H)-dione **3f**

Yield 8.6 g (60% method A). Yield 8.9 g (62% method B). Yield 12.2 g (85% method B). Colorless powder: mp 73–74 °C. ^1^H-NMR (300 MHz, CDCl_3_): δ 1.69 (s, 6H, 2CH_3_), 4.35 (s, 2H, CH_2_), 7.60–7.67 (m, 2H, arom H), 7.70–7.77 (m, 2H, arom H). ^13^C-NMR (125 MHz, CDCl_3_): δ 24.8 (s, 2CH_3_), 58.3 (s, C), 70.7 (s, CH_2_), 120.2 (q, *J*_CF_ = 253.3 Hz, CF_3_), 122.5 (s, arom. C), 131.3 (s, arom. C), 133.6 (s, arom. C), 168.9 (s, C=O). ^19^F-NMR (376.5 MHz, CDCl_3_): δ −61.11 (s, CF_3_). LC-MS, *m*/*z*: 288 [M + H]^+^. Anal. calcd for C_13_H_12_F_3_NO_3_: C, 54.36; H, 4.21; N, 4.88; found: C, 54.47; H, 4.47; N, 4.85.

#### 3.6.7. 2-[2-(Trifluoromethoxy)propyl]-1*H*-isoindole-1,3(2*H*)-dione **3g**

Yield 3.1 g (23% method A). Yield 4.6 g (34% method B). Yield 13.5 g (99% method B). Colorless powder: mp 71–72 °C. ^1^H-NMR (500 MHz, CDCl_3_): δ 1.40–1.43 (m, 3H, CH_3_), 3.79 (dd, ^2^*J* = 14.0 Hz, ^3^*J* = 2.0 Hz, 1H, CH_2_), 3.94–4.00 (m, 1H, CH_2_), 4.80–4.95 (m, 1H, CH), 7.74 (s, 2H, arom H), 7.87 (s, 2H, arom H). ^13^C-NMR (125 MHz, CDCl_3_): δ 18.6 (s, CH_3_), 42.3 (s, CH_2_), 72.9 (s, C), 121.5 (q, *J*_CF_ = 255.1 Hz, CF_3_), 123.5 (s, arom. C), 131.7 (s, arom. C), 134.2 (s, arom. C), 168.0 (s, C=O). ^19^F-NMR (376.5 MHz, CDCl_3_): δ −59.19 (s, CF_3_). LC-MS, *m*/*z*: 274 [M + H]^+^. Anal. calcd for C_12_H_10_F_3_NO_3_: C, 52.75; H, 3.69; N, 5.13; found: C, 52.70; H, 3.44; N, 4.12.

## 4. Conclusions

The “chlorination/fluorination” technique for aromatic trifluoromethyl ether syntheses, since 1955, has been a powerful driver of research in organofluorine chemistry. More than 30,000 substances [4] with such a group were prepared in the last half century. It was shown that this method, as well as oxidative desulfurization-fluorination, can be successfully applied for a wide range of aliphatic substrates with various functional groups. It requires cheap reagents and it is promising in industrial applications.

## Figures and Tables

**Table 1:** Structures and yields of chlorinated and fluorinated products.

Entry	Chlorinated Product	Yield%	Fluorinated Product	Yield %		
				Method A	Method B	Method C
1	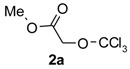	87	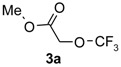	traces	traces	36
2	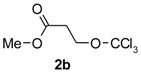	89	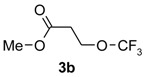	53	69	63
3	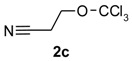	92	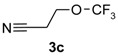	22	66	66
4	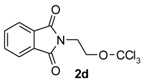	96	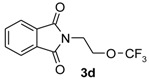	90	93	96
5	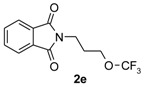	95	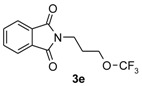	88	90	95
6	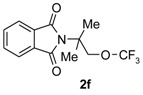	82	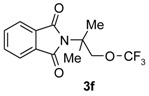	60	62	85
7	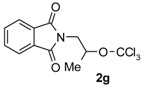	85	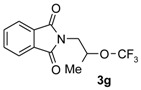	23	34	99

Note: Method A: SbF3 with SbCl5 (cat.), method B: HF with SbCl5 (cat.), method C: Py / HF70 %, DBH.

## Supplementary Materials

^1^H-, ^13^C-, and ^19^F-NMR spectra of products associated with this article are available online.
