# Supplementary material for: Evaluation of Efficient and Practical Methods for the Preparation of Functionalized Aliphatic Trifluoromethyl Ethers
Source: Molecules. 2017 May 14;22(5):804. doi: 10.3390/molecules22050804 (PMC6154681; doi:10.3390/molecules22050804)
Supplement: Supplementary file 1 [file molecules-22-00804-s001.pdf]

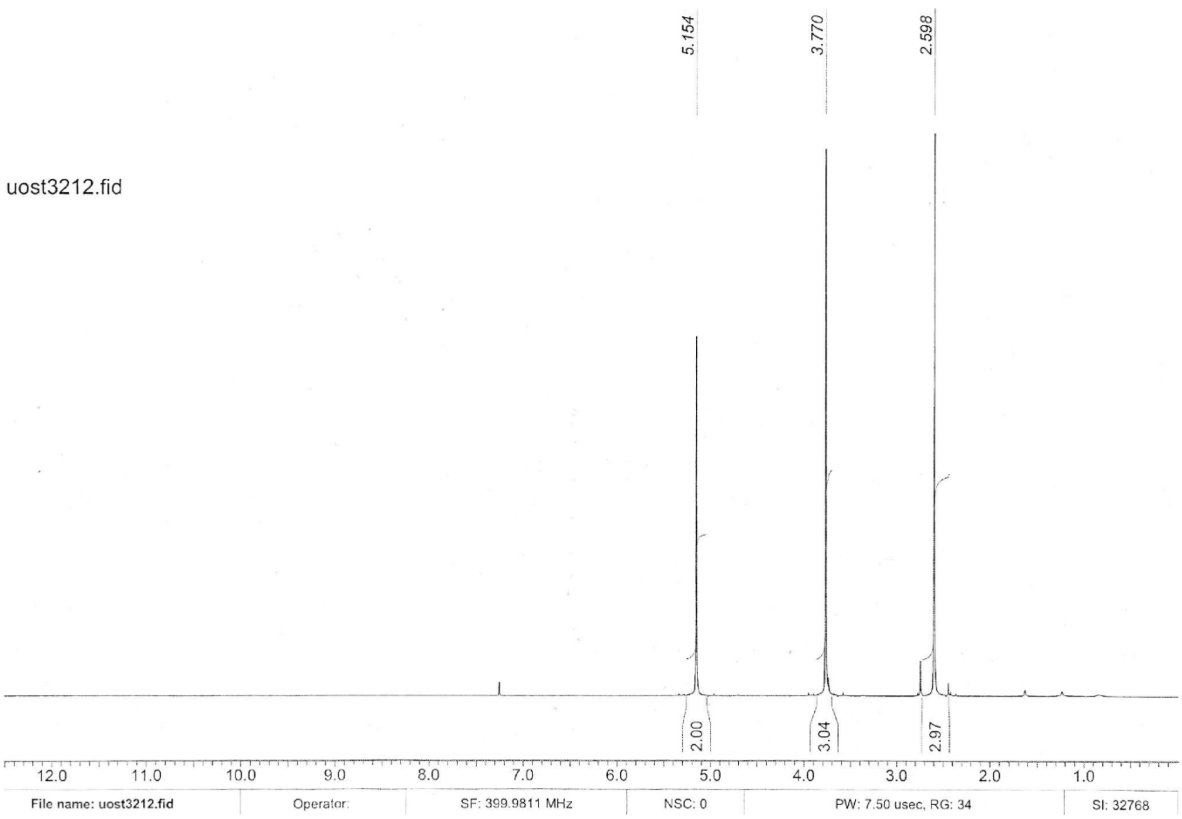

Figure S1. ^{1}H-NMR spectra of methyl ([methylthio]carbonothioyl)oxy)acetate **1a**.

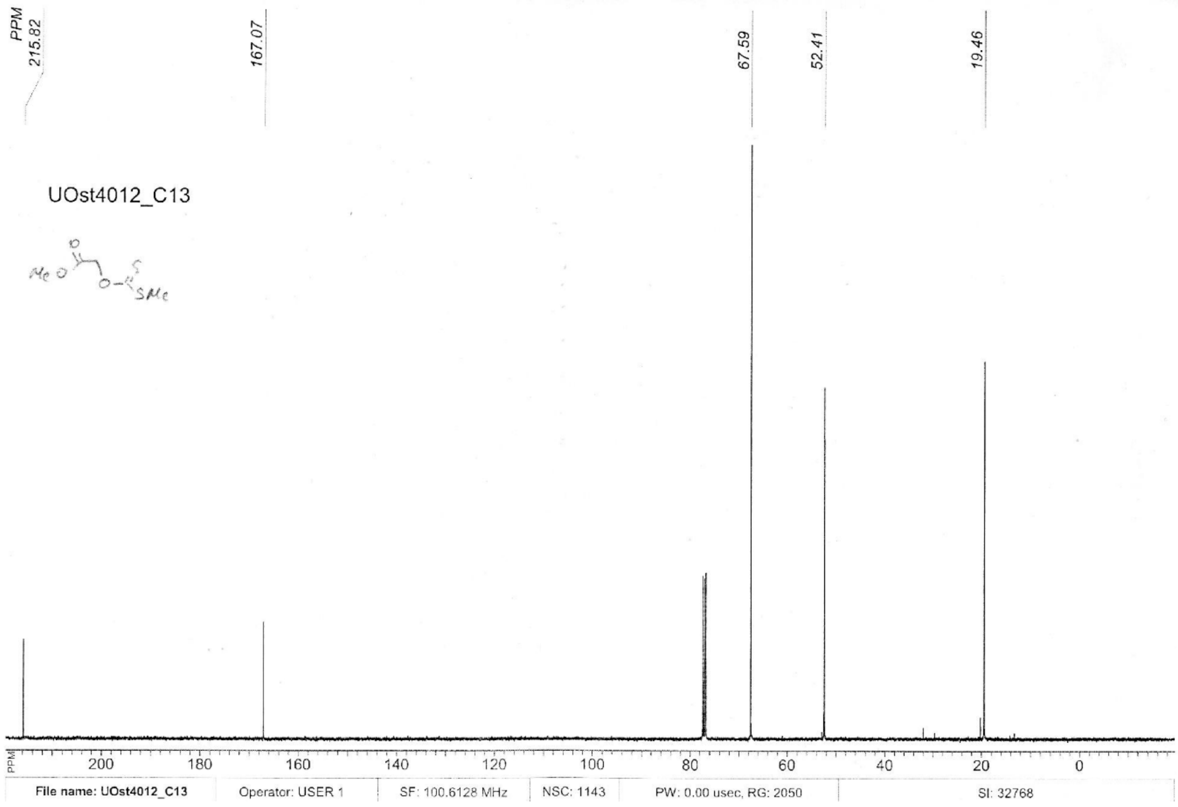

Figure S2. ^{13}C-NMR spectra of methyl ([(methylthio)carbonothioyl]oxy)acetate **1a**.

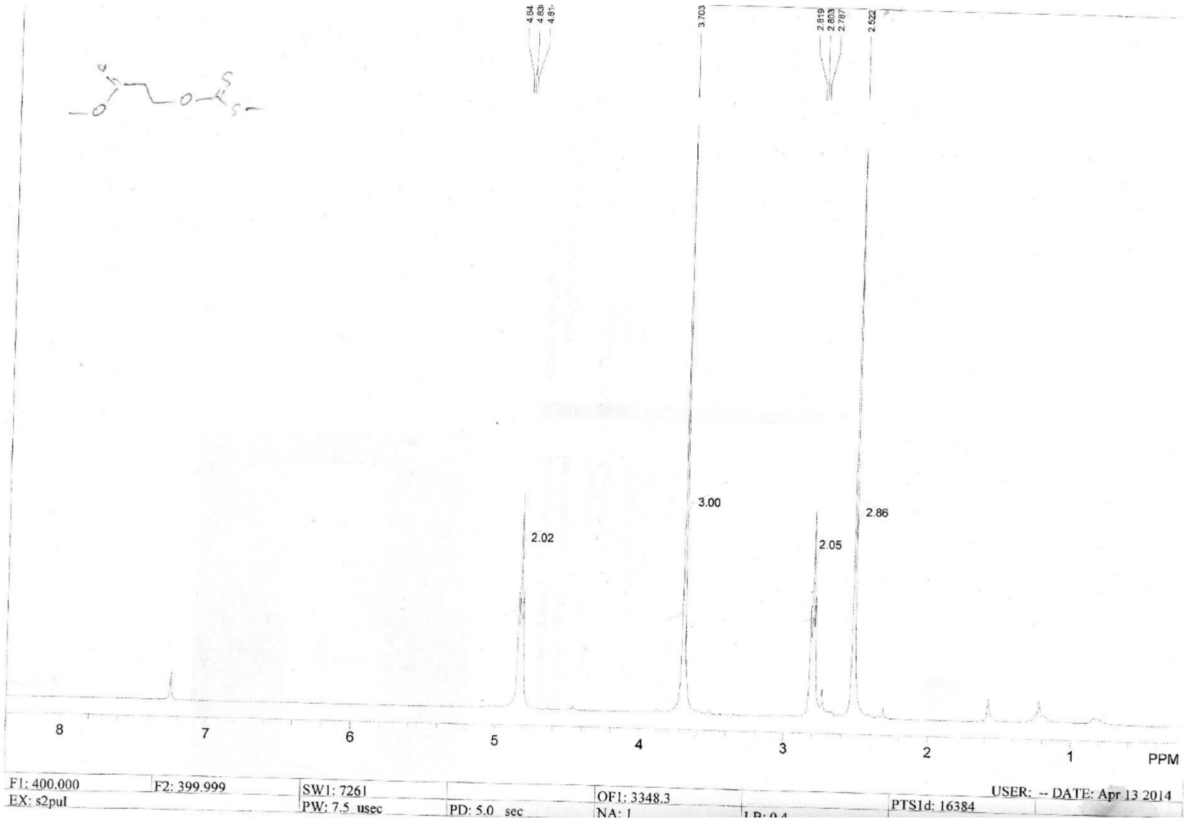

Figure S3. ^{1}H-NMR spectra of methyl 3-([(methylsulfanyl)carbonothioyl]oxy)propanoate **1b**.

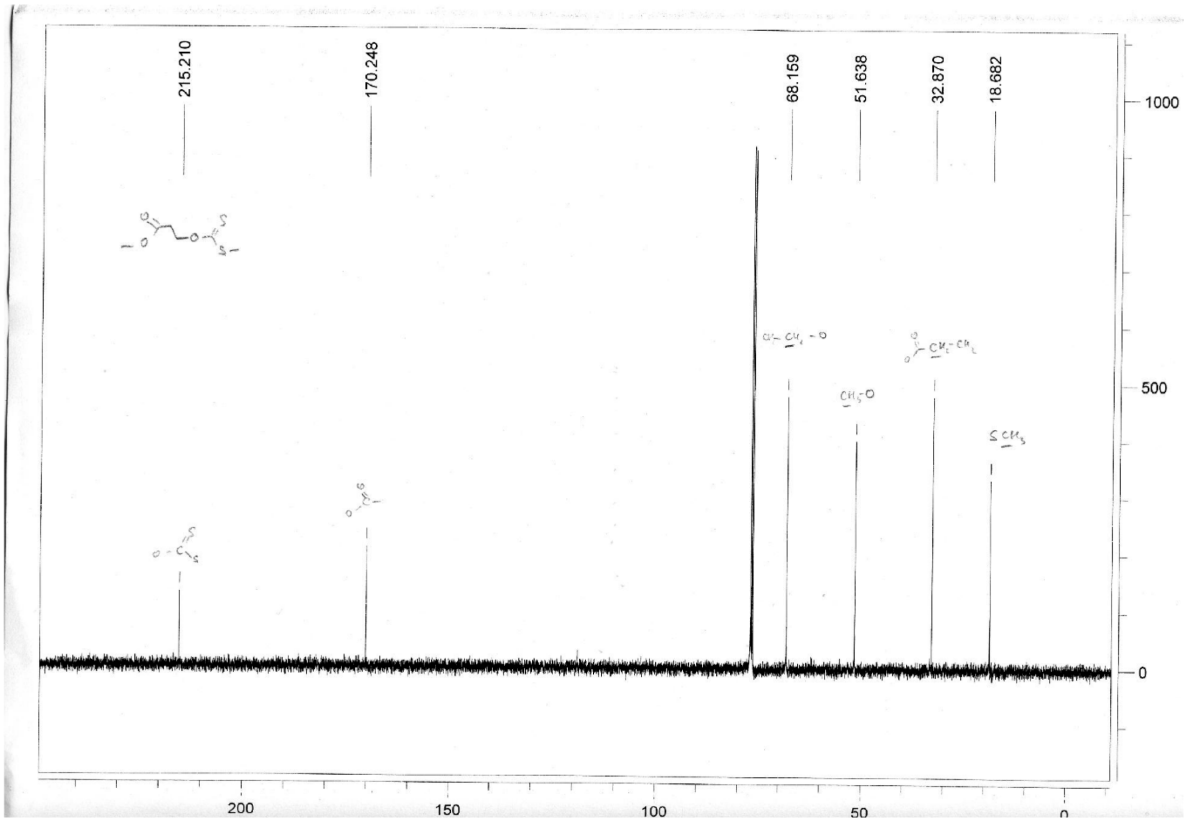

Figure S4. ^{13}C-NMR spectra of methyl 3-([(methylsulfanyl)carbonothioyl]oxy)propanoate **1b**.

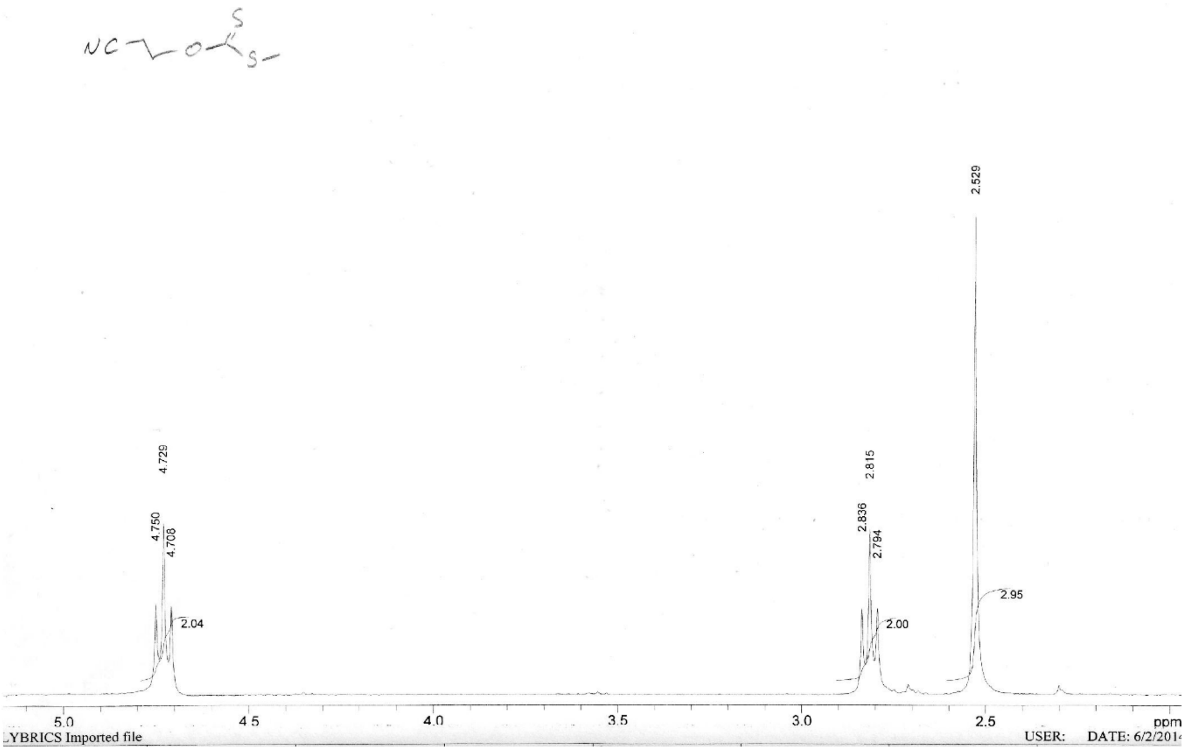

Figure S5. ^{1}H-NMR spectra of O-(2-cyanoethyl)-S-methyl-dithiocarbonate **1c**.

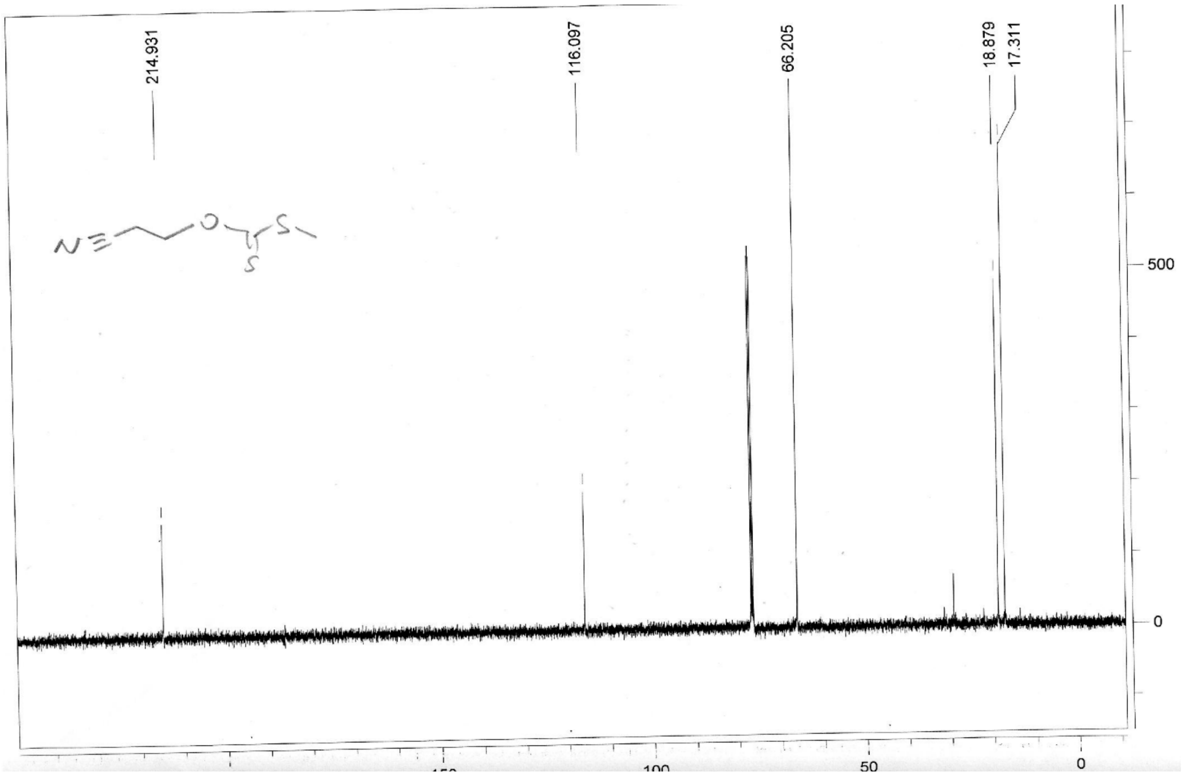

Figure S6. ^{13}C-NMR spectra of O-(2-cyanoethyl)-S-methyl-dithiocarbonate **1c**.

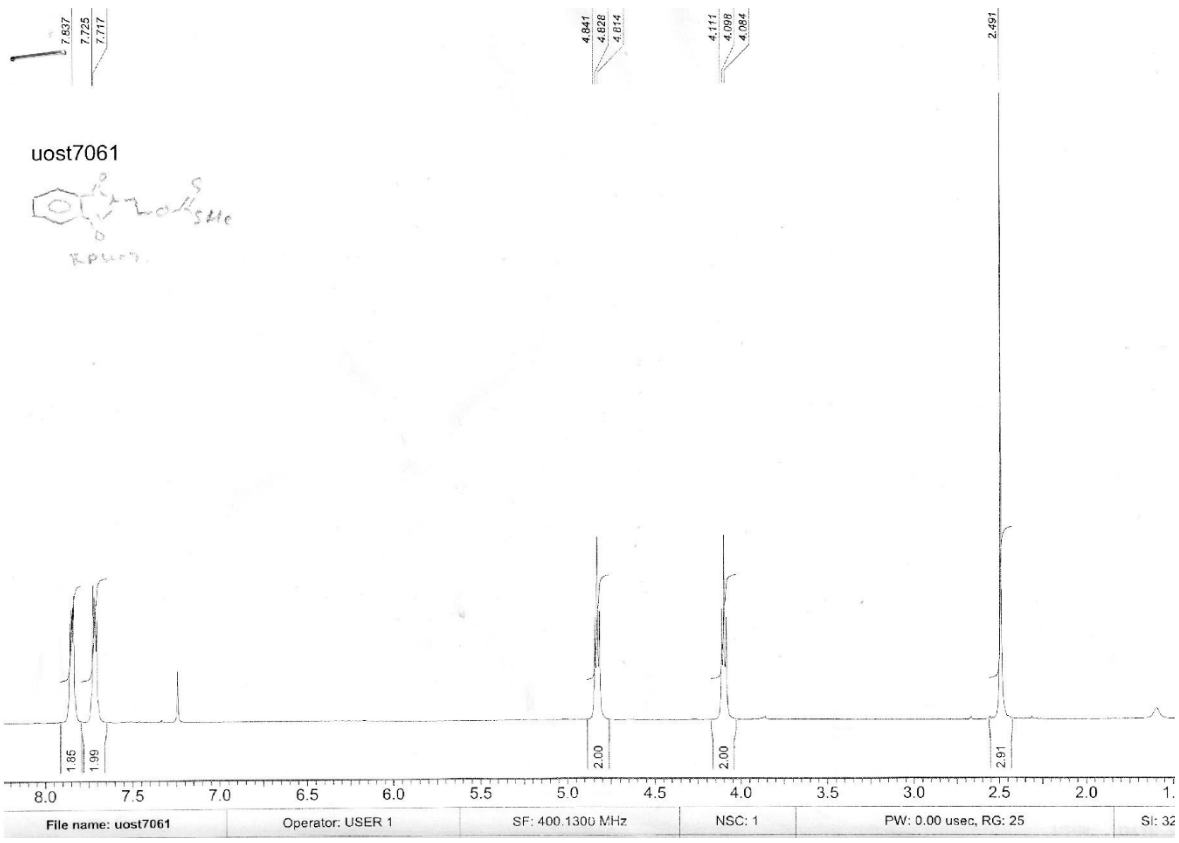

Figure S7. ^{1}H-NMR spectra of O-[2-(1,3-dioxo-1,3-dihydro-2H-isoindol-2-yl)ethyl] S-methyldithiocarbonate **1d**.

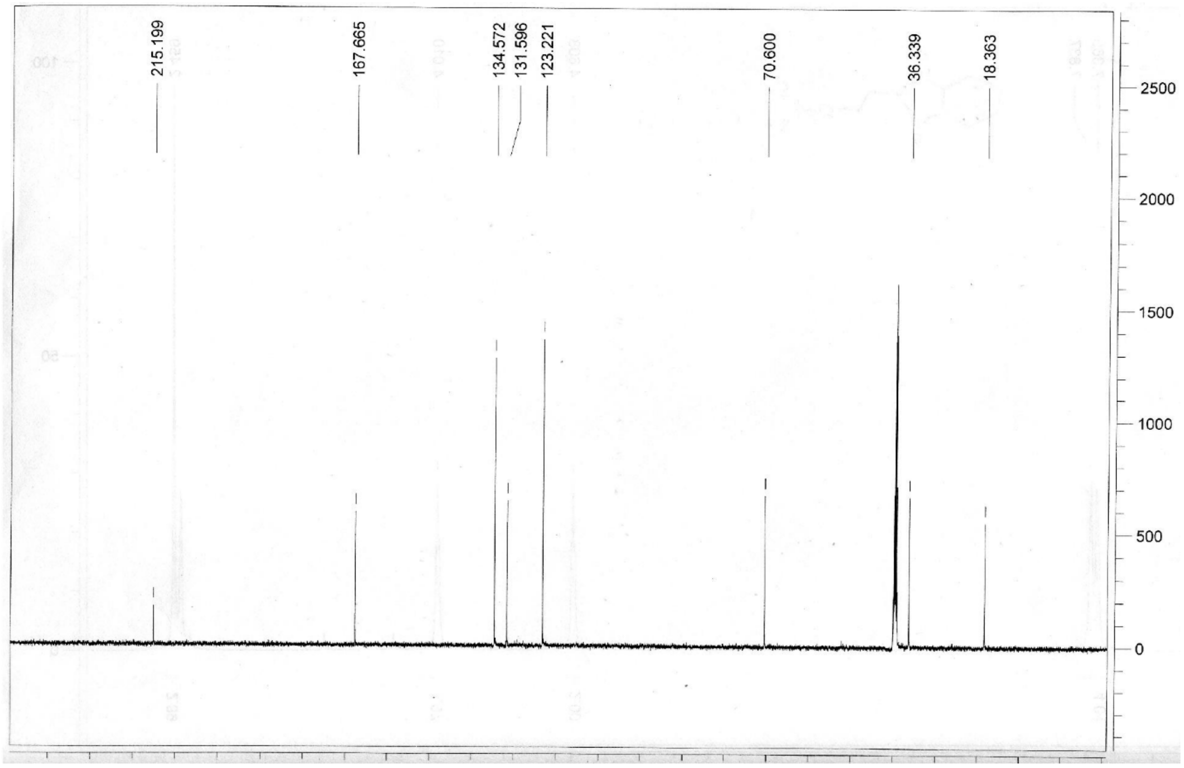

Figure S8. ^{13}C-NMR spectra of O-[2-(1,3-dioxo-1,3-dihydro-2H-isoindol-2-yl)ethyl] S-methyldithiocarbonate **1d**.

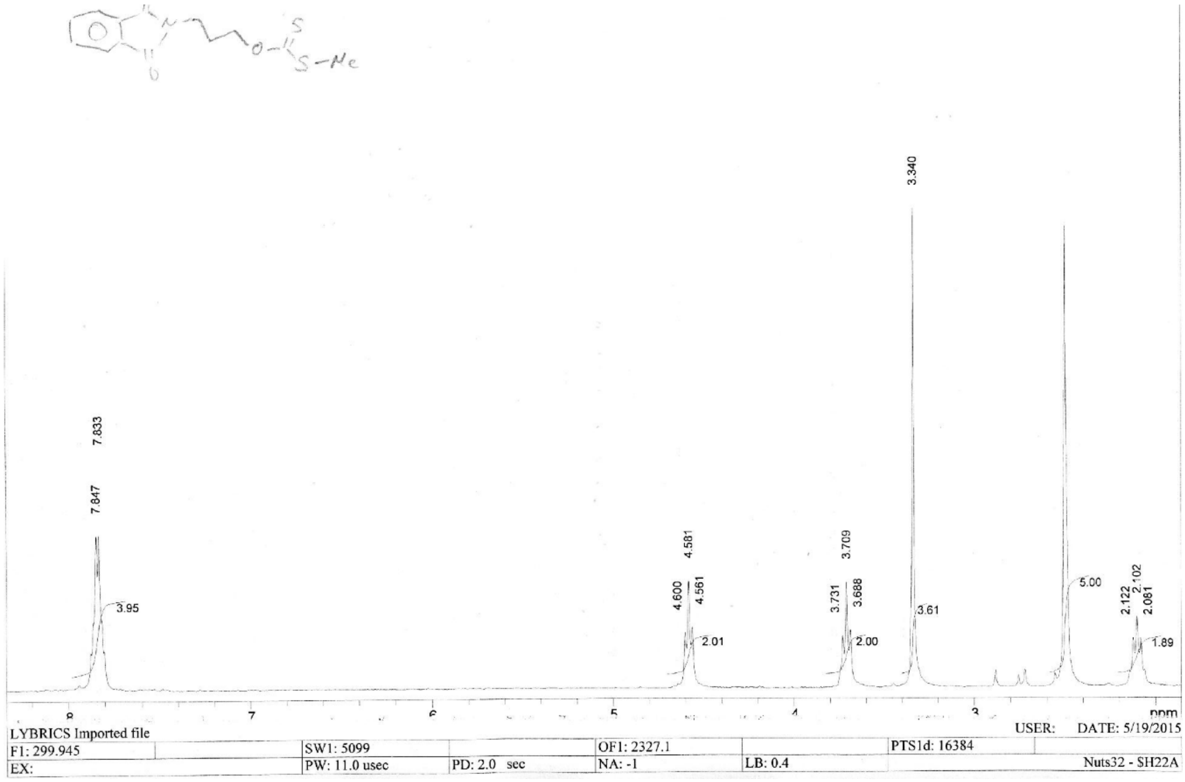

Figure S9. ^{1}H-NMR spectra of O-[3-(1,3-dioxo-1,3-dihydro-2H-isoindol-2-yl)propyl] S-methyldithiocarbonate **1e**.

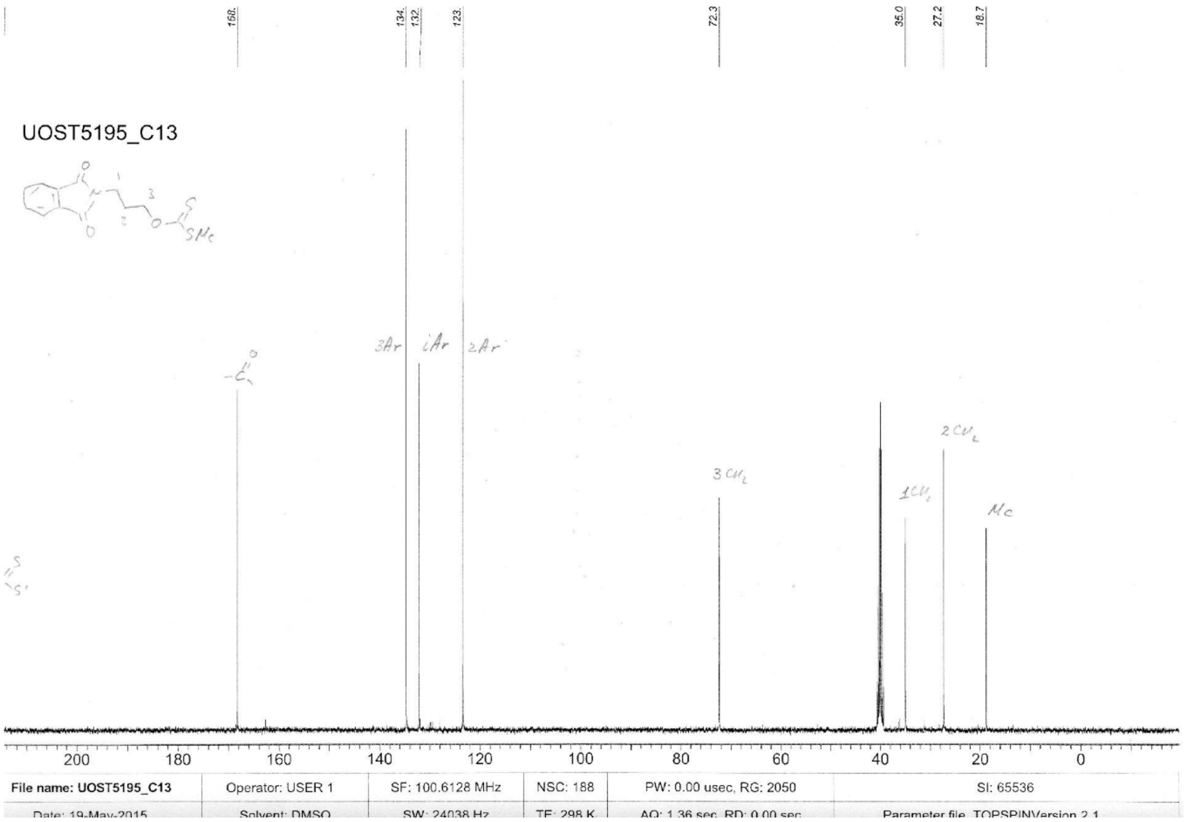

Figure S10. ^{13}C-NMR spectra of O-[3-(1,3-dioxo-1,3-dihydro-2H-isoindol-2-yl)propyl] S-methyldithiocarbonate **1e**.

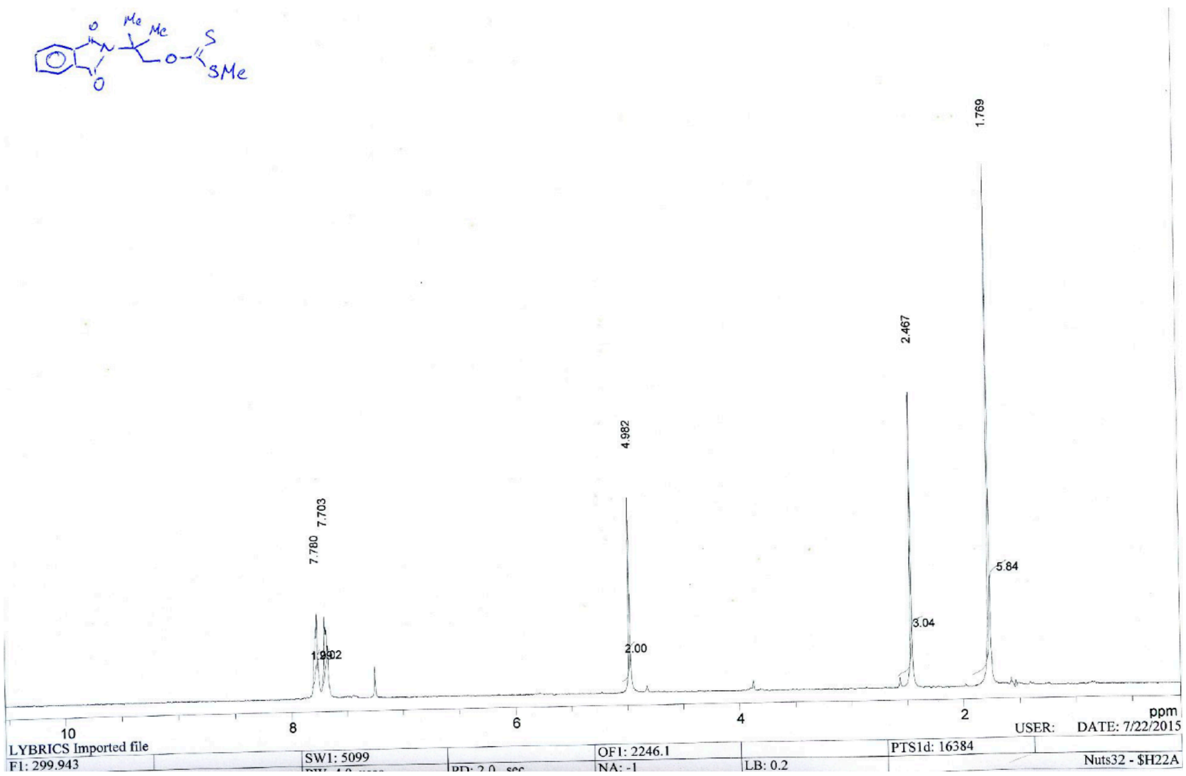

Figure S11. ^{1}H-NMR spectra of O-[2-(1,3-dioxo-1,3-dihydro-2H-isoindol-2-yl)-2-methylpropyl] S-methyldithiocarbonate **1f**.

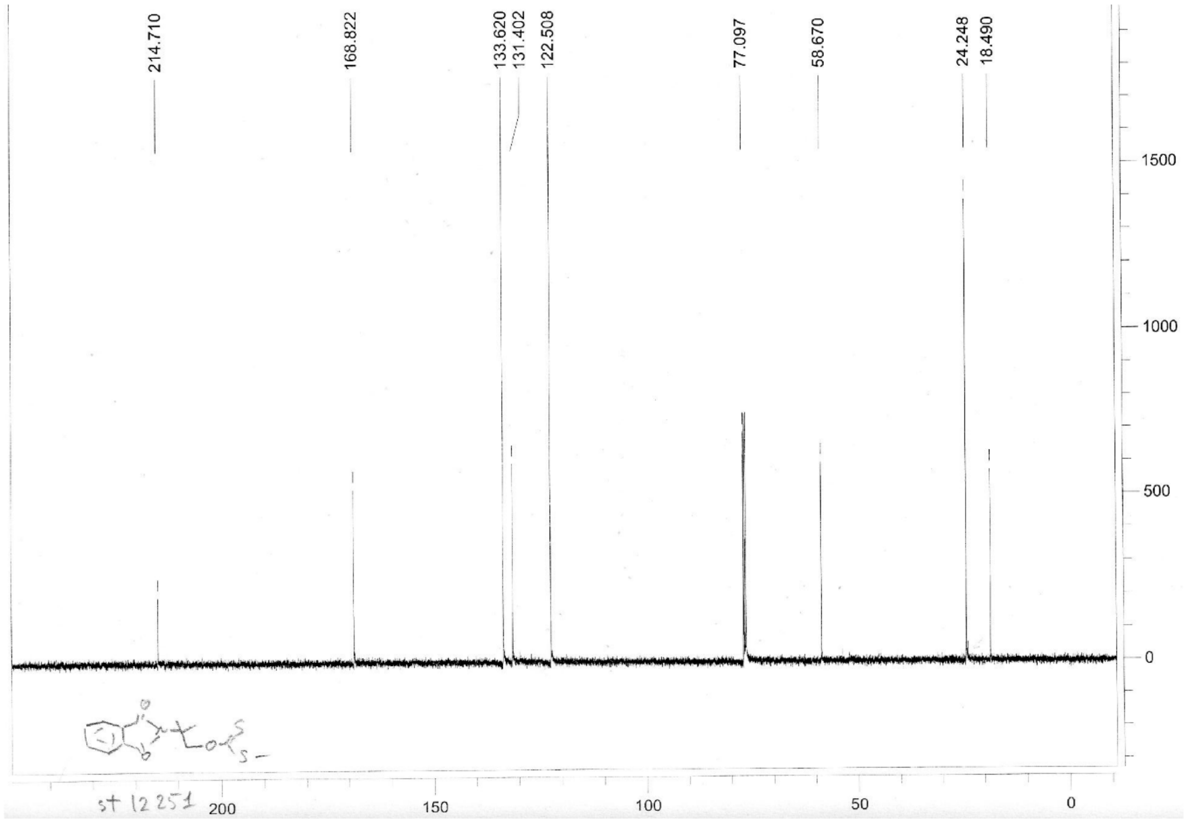

Figure S12. ^{13}C-NMR spectra of O-[2-(1,3-dioxo-1,3-dihydro-2H-isoindol-2-yl)-2-methylpropyl] S-methyldithiocarbonate **1f**.

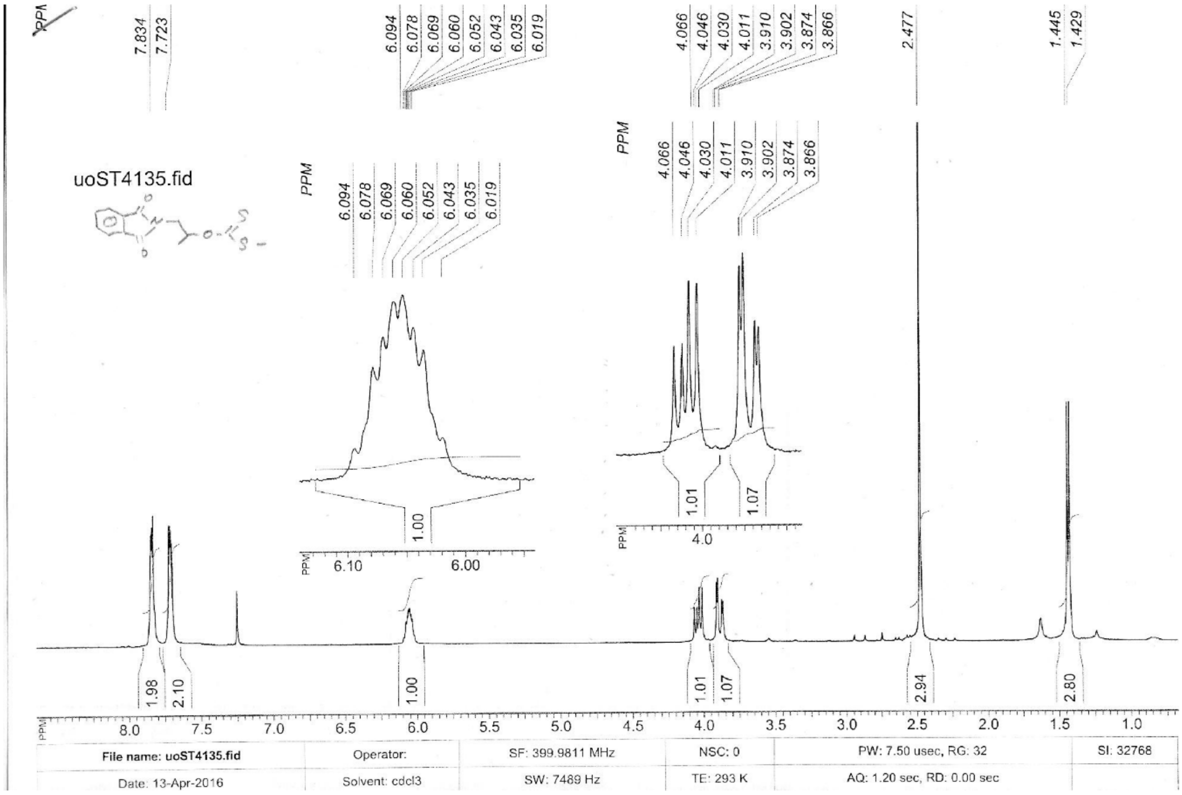

Figure S13. ^{1}H-NMR spectra of O-[2-(1,3-dioxo-1,3-dihydro-2H-isoindol-2-yl)-1-methylethyl] S-methyldithiocarbonate **1g**.

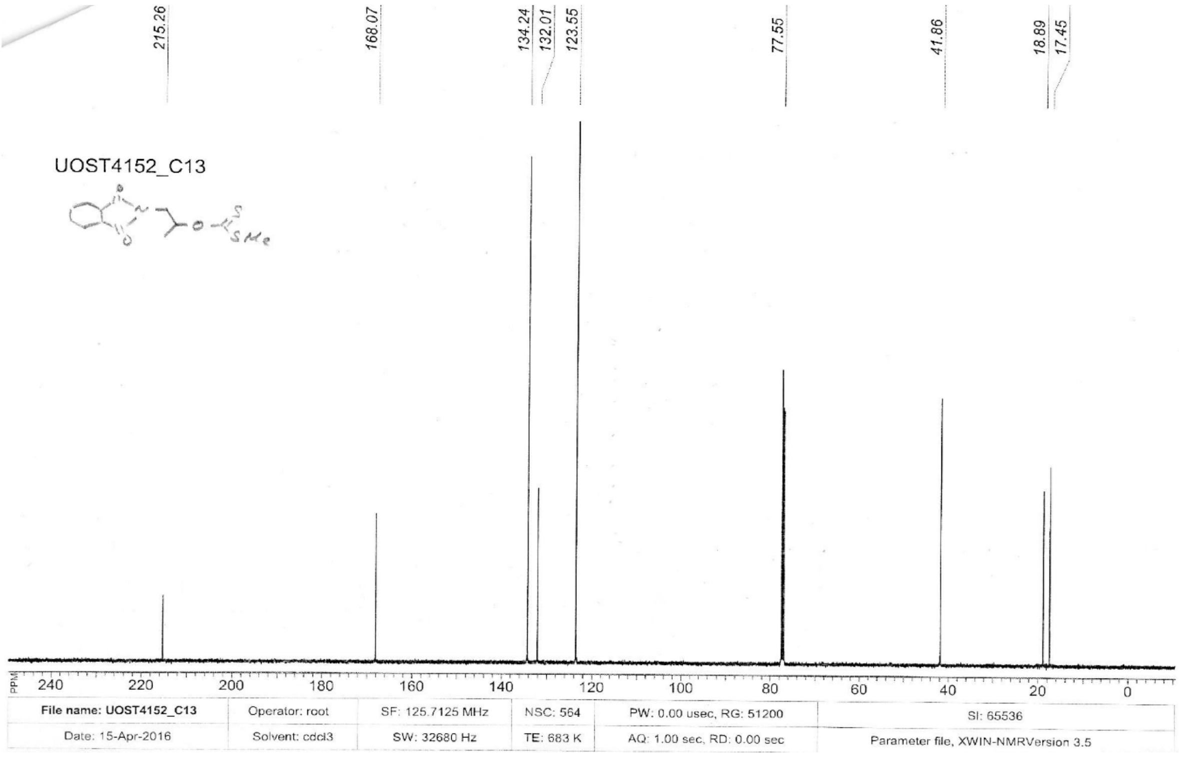

Figure S14. ^{13}C-NMR spectra of O-[2-(1,3-dioxo-1,3-dihydro-2H-isoindol-2-yl)-1-methylethyl] S-methyldithiocarbonate **1g**.

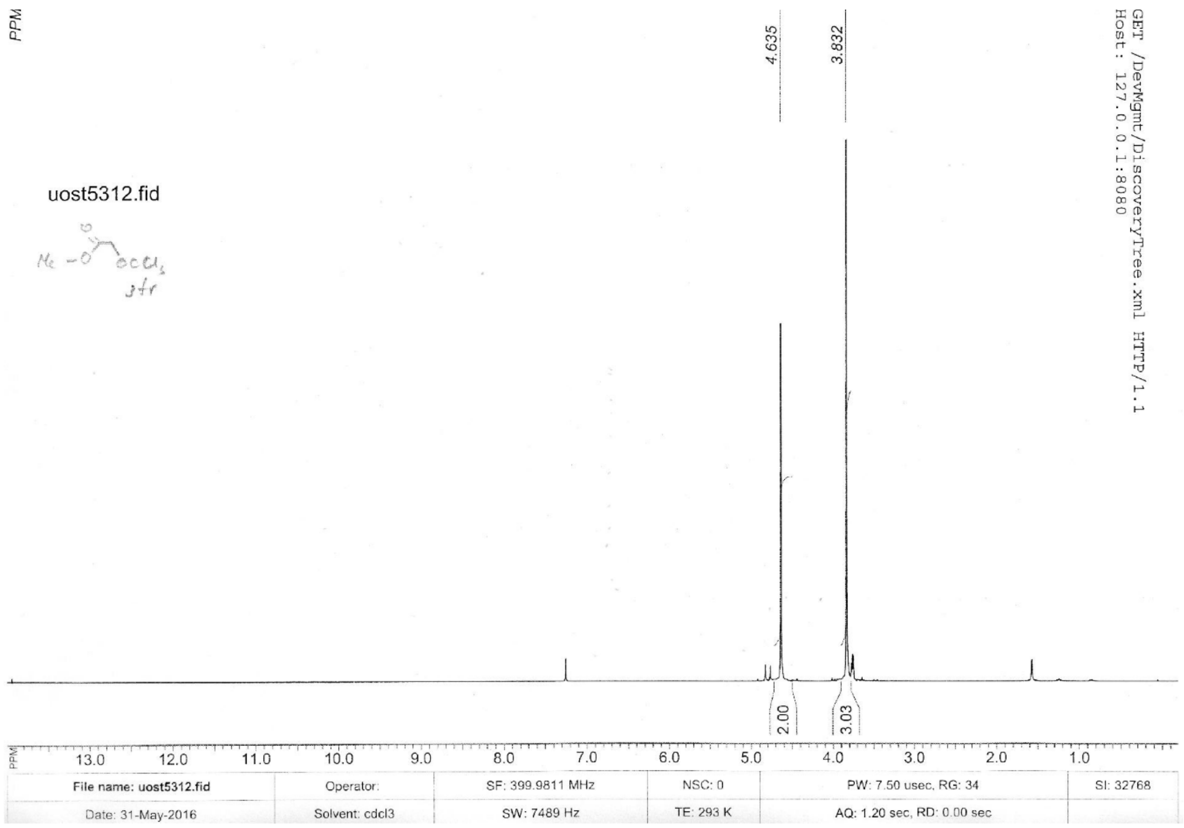

Figure S15. ^{1}H-NMR spectra of methyl (trichloromethoxy)acetate **2a**.

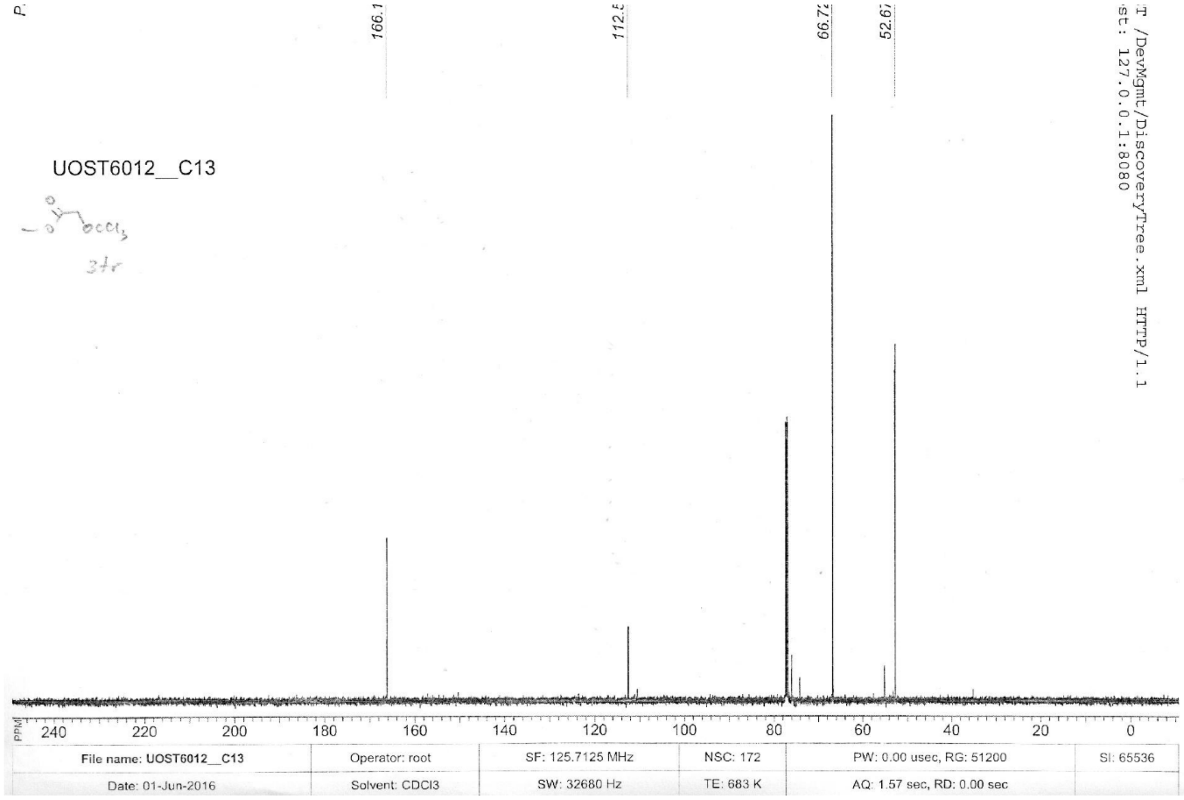

Figure S16. ^{13}C-NMR spectra of methyl (trichloromethoxy)acetate **2a**.

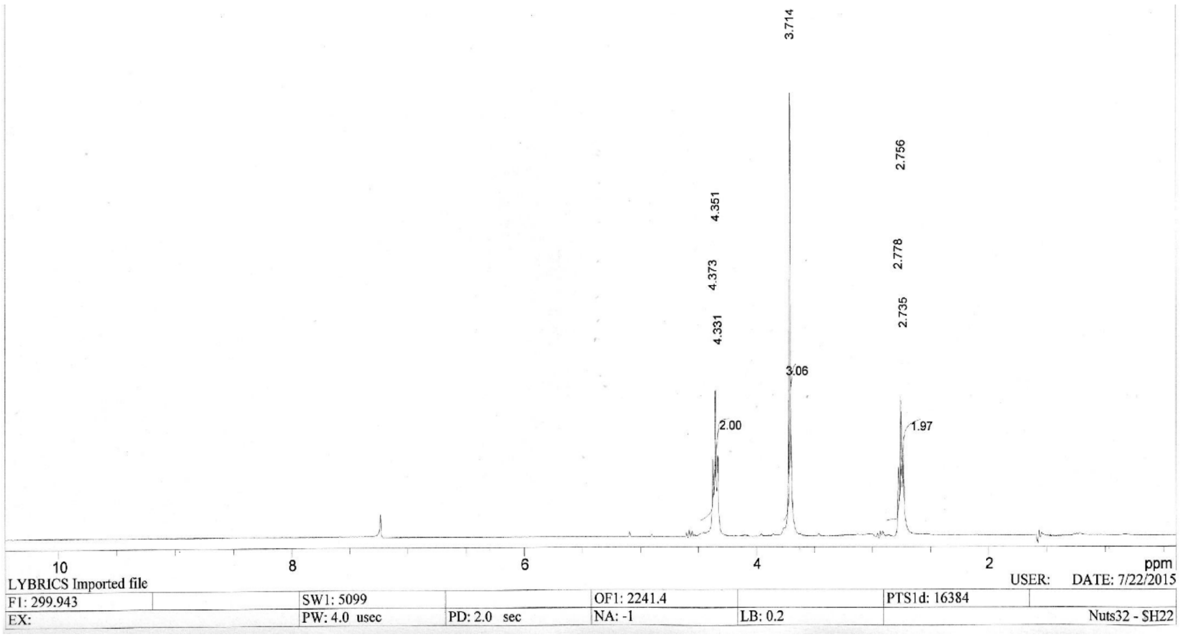

Figure S17. ^{1}H-NMR spectra of methyl 3-(trichloromethoxy)propanoate **2b**.

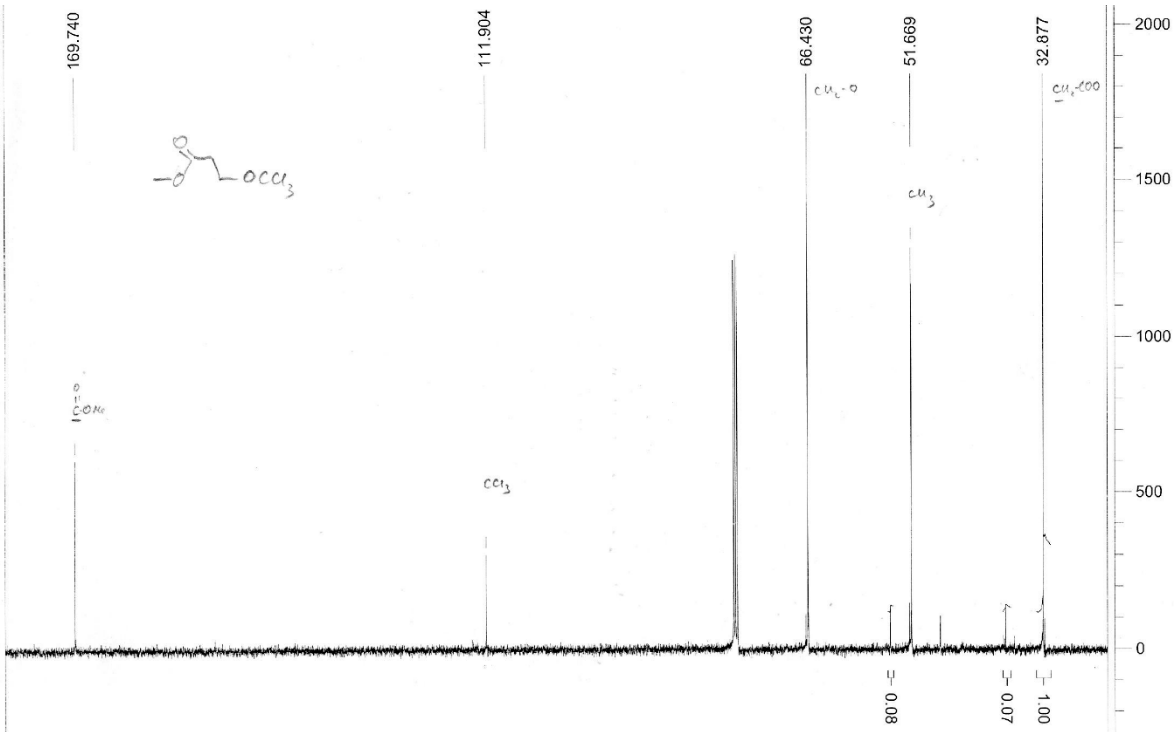

Figure S18. ^{13}C-NMR spectra of methyl 3-(trichloromethoxy)propanoate **2b**.

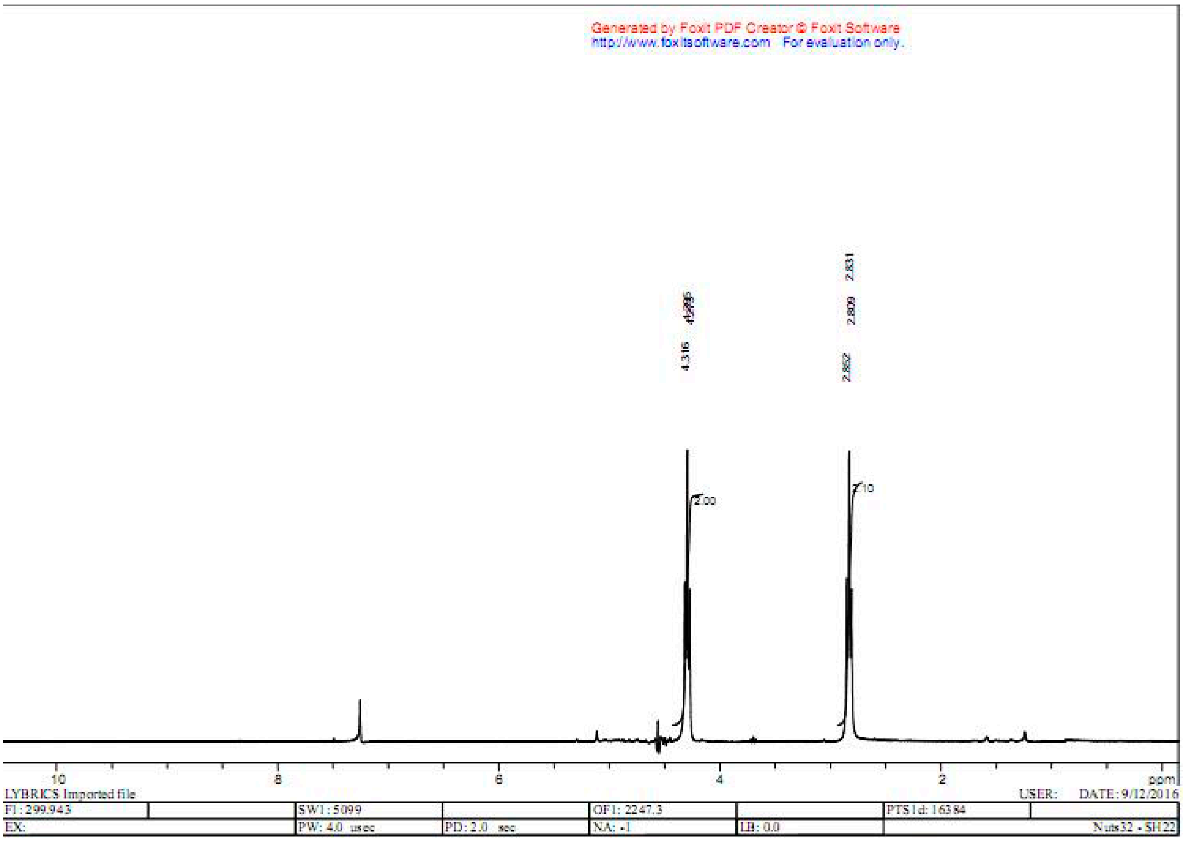

Figure S19. ^{1}H-NMR spectra of 3-(trichloromethoxy)propanenitrile **2c**.

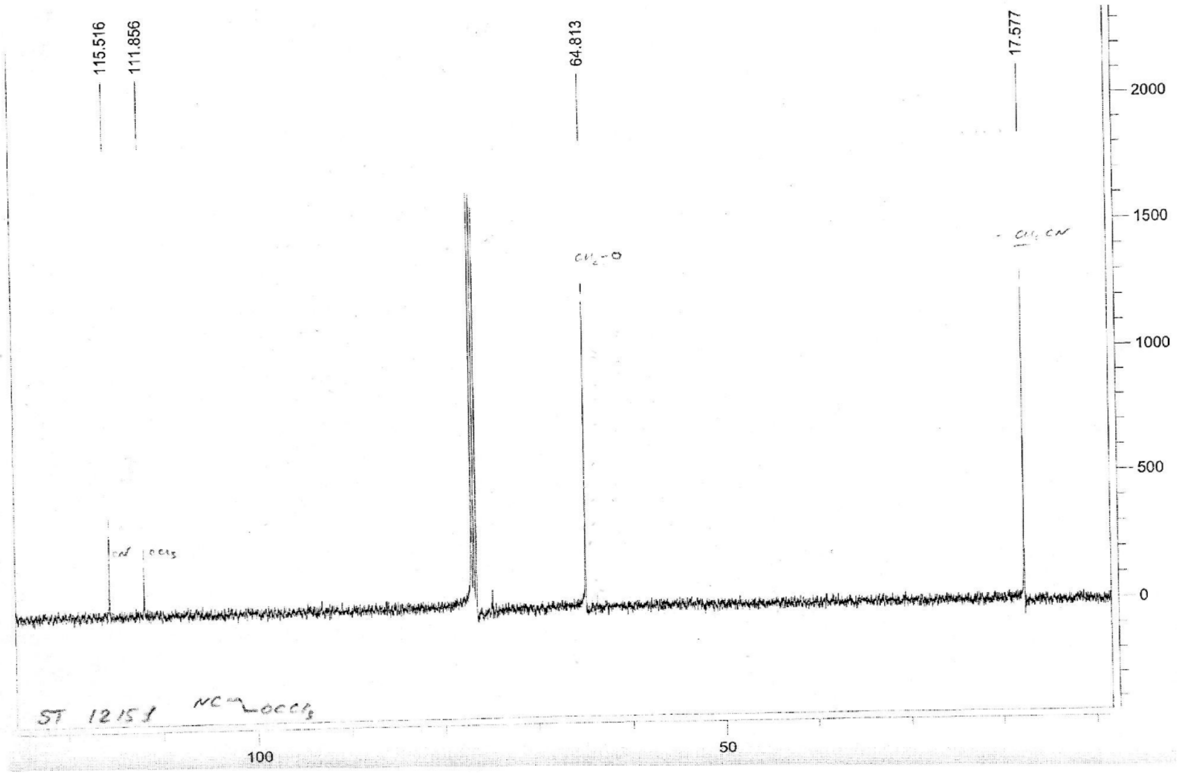

Figure S20. ^{13}C-NMR spectra of 3-(trichloromethoxy)propanenitrile **2c**.

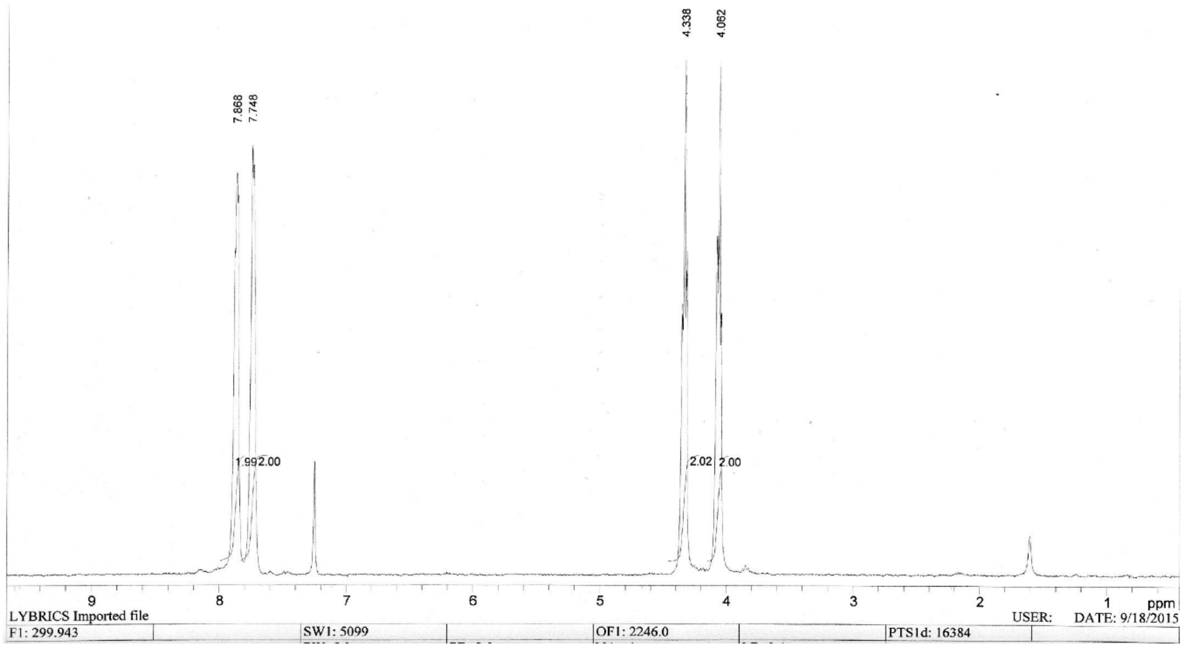

Figure S21. ^{1}H-NMR spectra of 2-[2-(trichloromethoxy)ethyl]-1H-isoindole-1,3(2H)-dione **2d**.

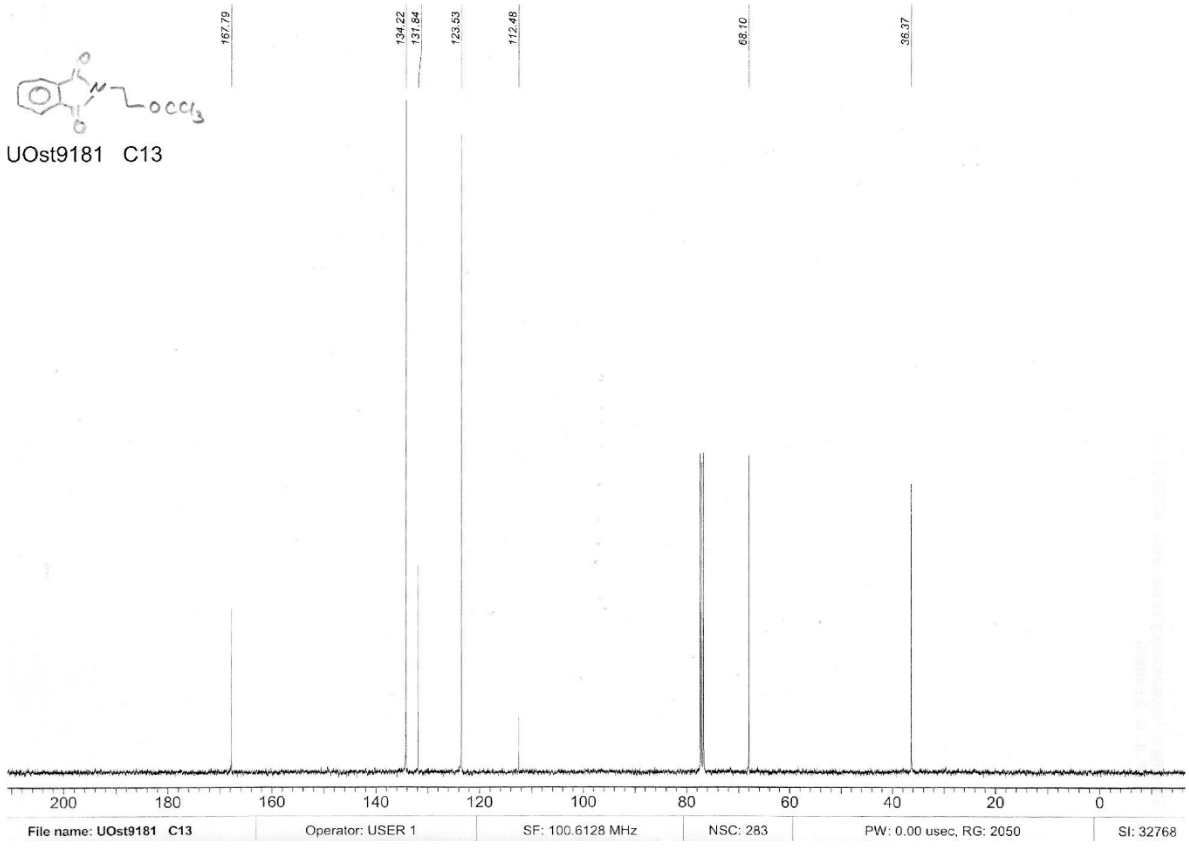

Figure S22. ^{13}C-NMR spectra of 2-[2-(trichloromethoxy)ethyl]-1H-isoindole-1,3(2H)-dione **2d**.

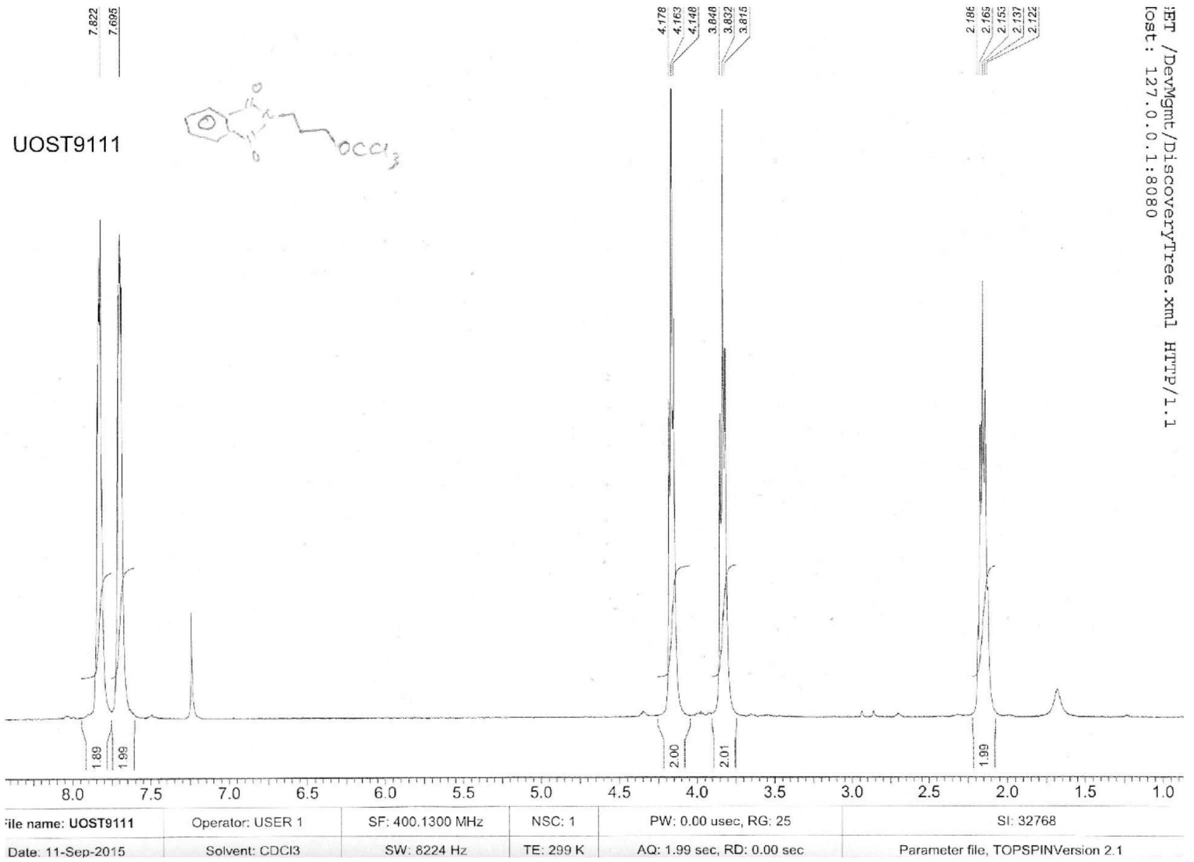

Figure S23. ^{1}H-NMR spectra of 2-[3-(trichloromethoxy)propyl]-1H-isoindole-1,3(2H)-dione **2e**.

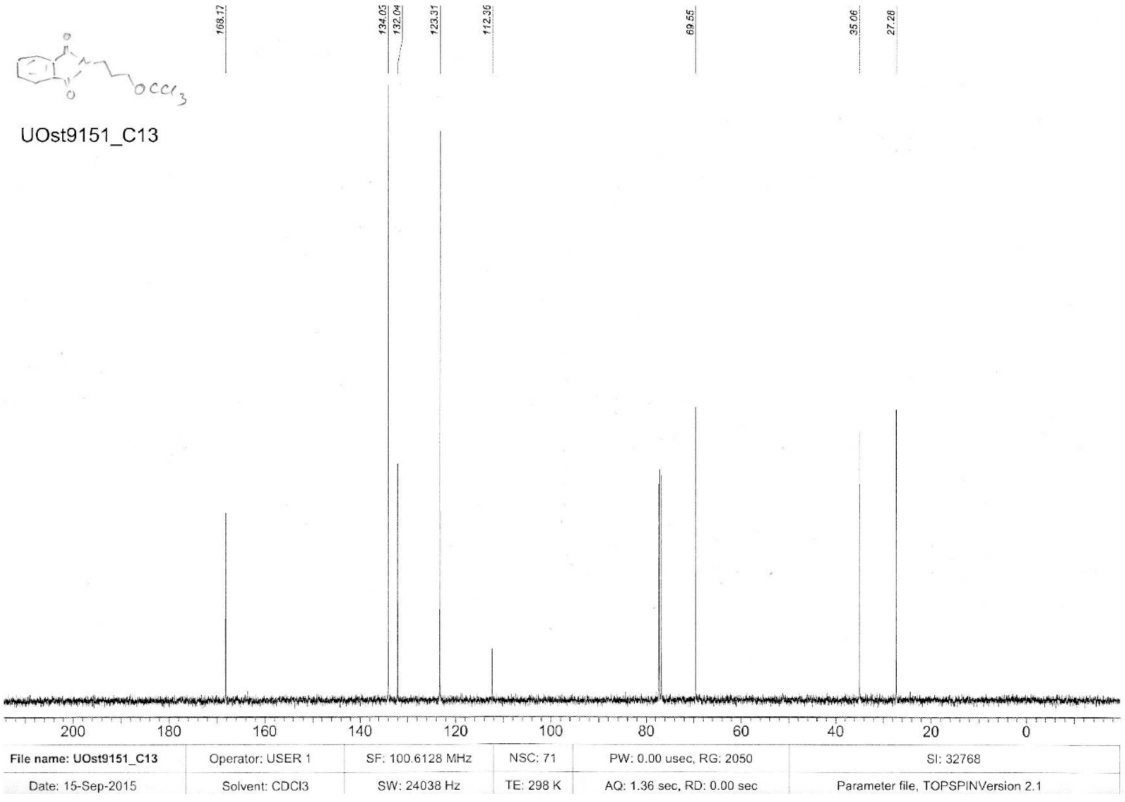

Figure S24. ^{13}C-NMR spectra of 2-[3-(trichloromethoxy)propyl]-1H-isoindole-1,3(2H)-dione **2e**.

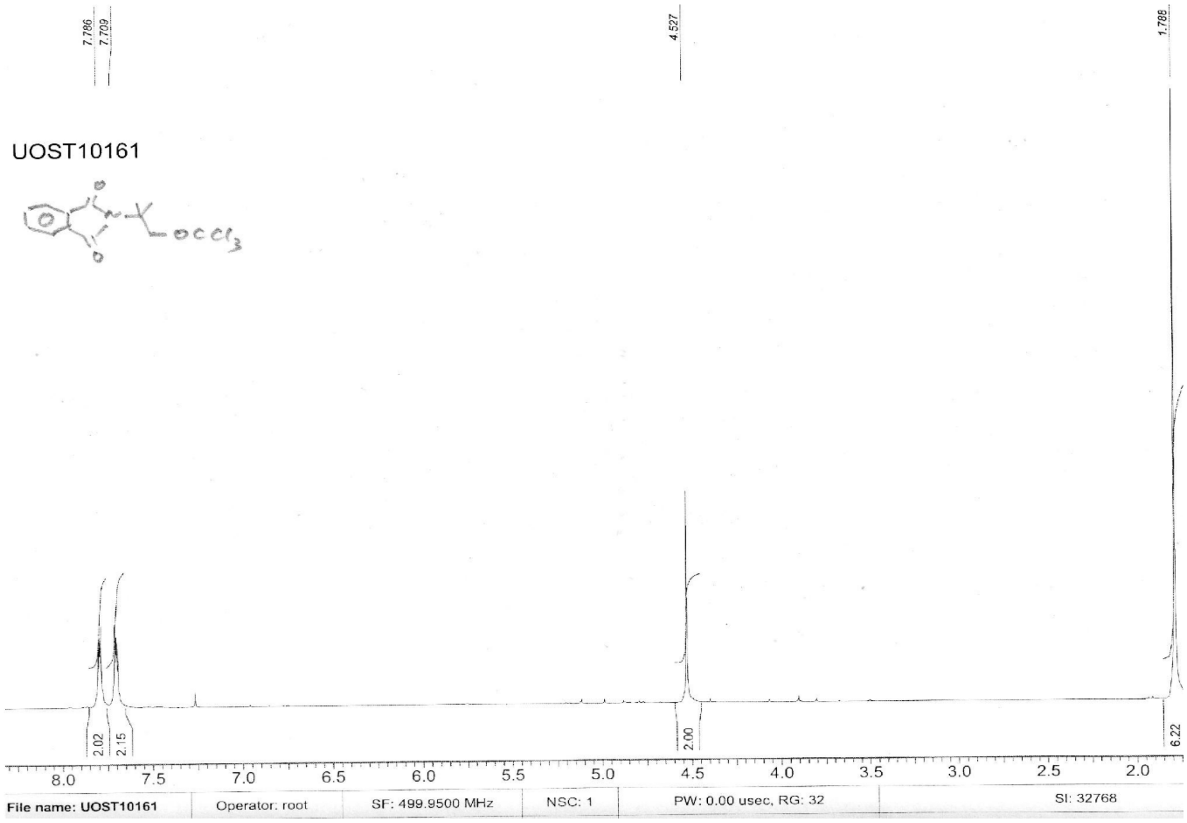

Figure S25. ^{1}H-NMR spectra of 2-[1,1-Dimethyl-2-(trichloromethoxy)ethyl]-1H-isoindole-1,3(2H)-dione **2f**.

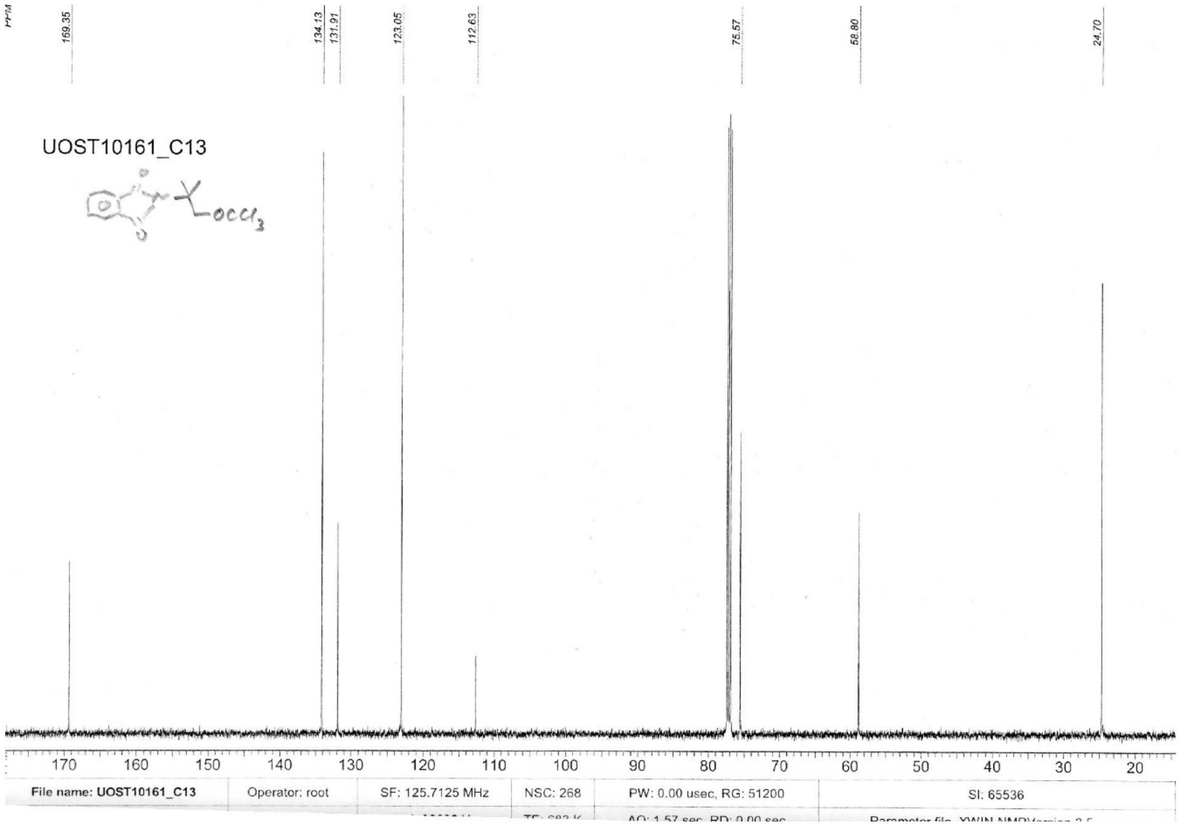

Figure S26. ^{13}C-NMR spectra of 2-[1,1-Dimethyl-2-(trichloromethoxy)ethyl]-1H-isoindole-1,3(2H)-dione **2f**.

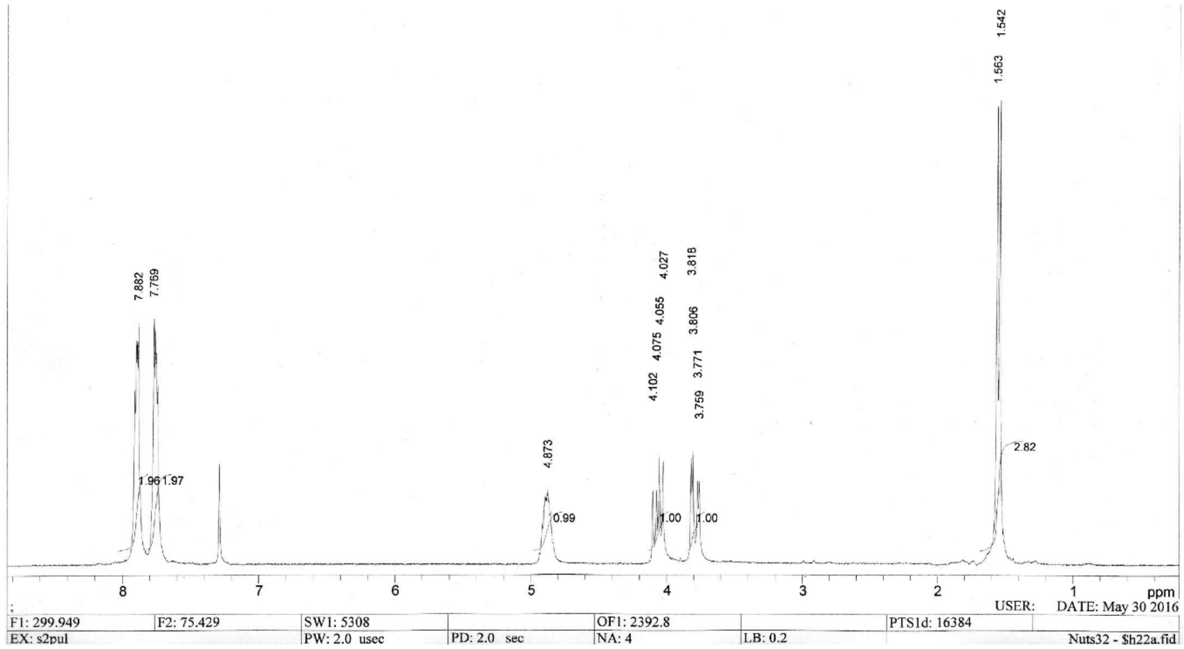

Figure S27. ^{1}H-NMR spectra of 2-[2-(Trichloromethoxy)propyl]-1H-isoindole-1,3(2H)-dione **2g**.

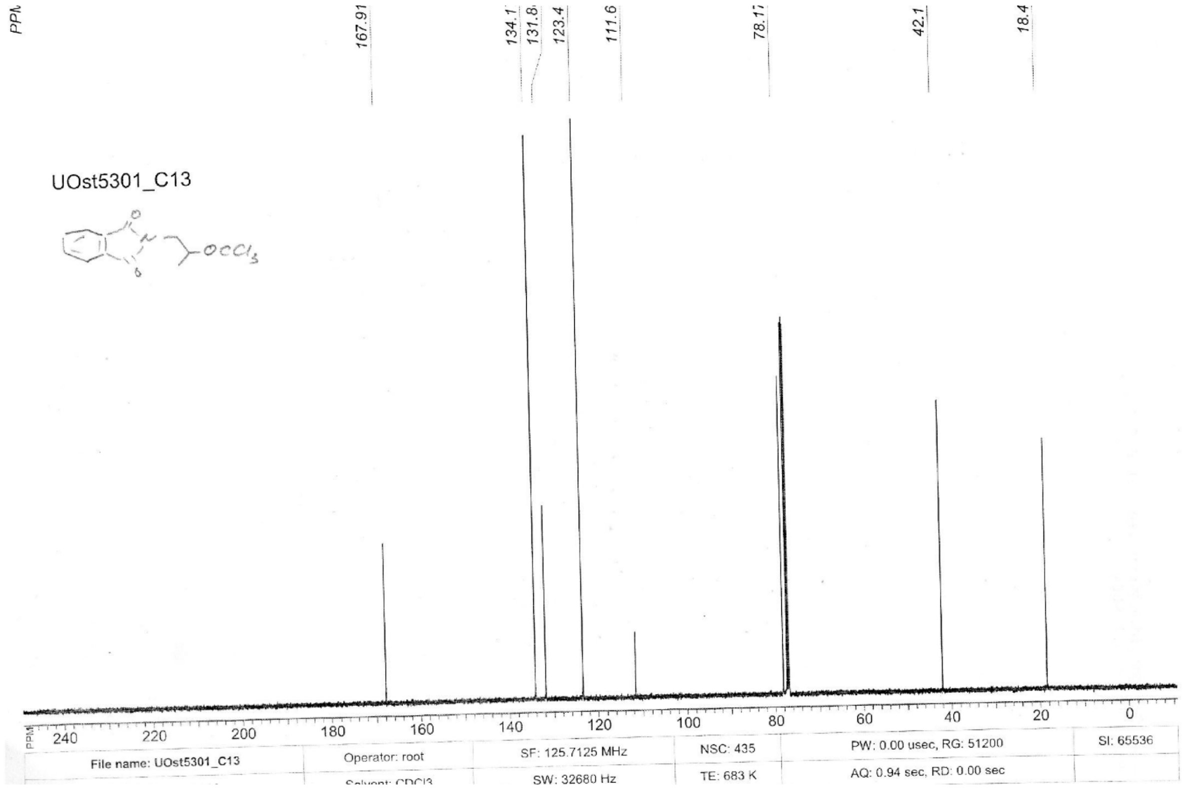

Figure S28. ^{13}C-NMR spectra of 2-[2-(Trichloromethoxy)propyl]-1H-isoindole-1,3(2H)-dione **2g**.

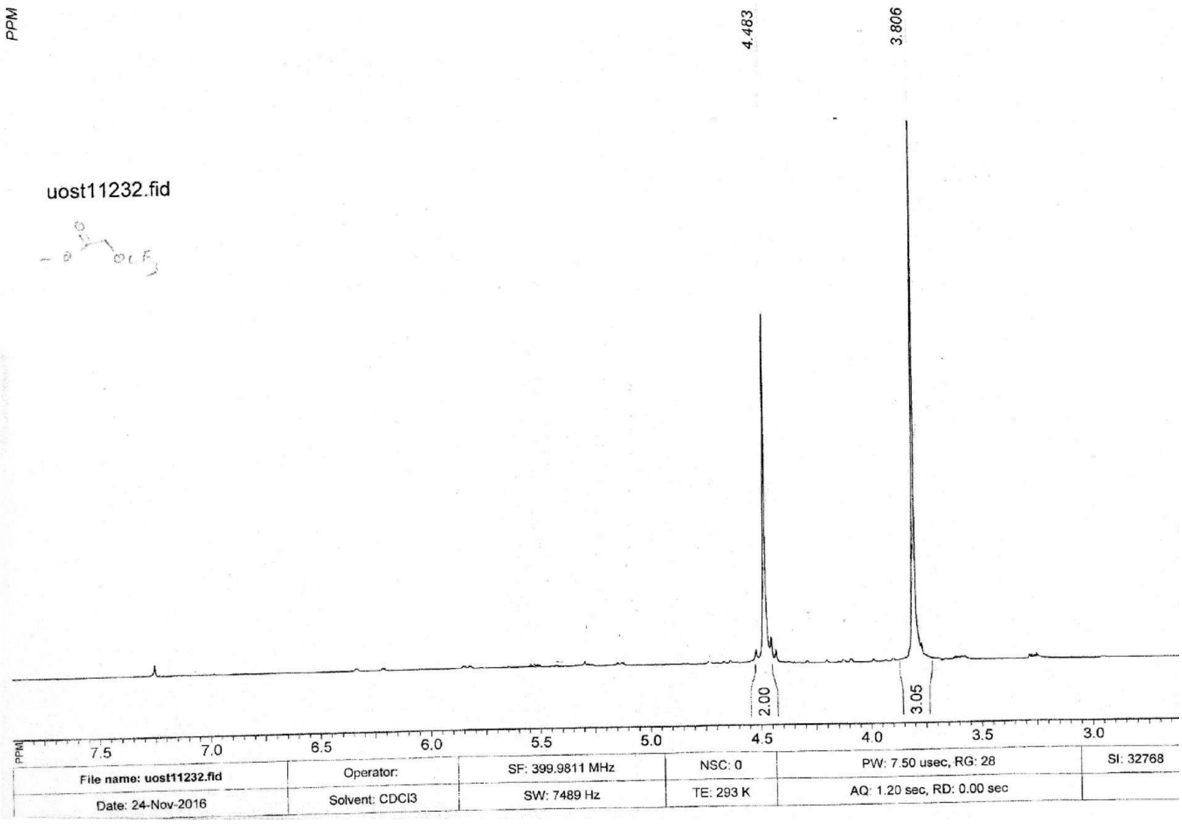

Figure S29. ^{1}H-NMR spectra of methyl (trifluoromethoxy)acetate **3a**.

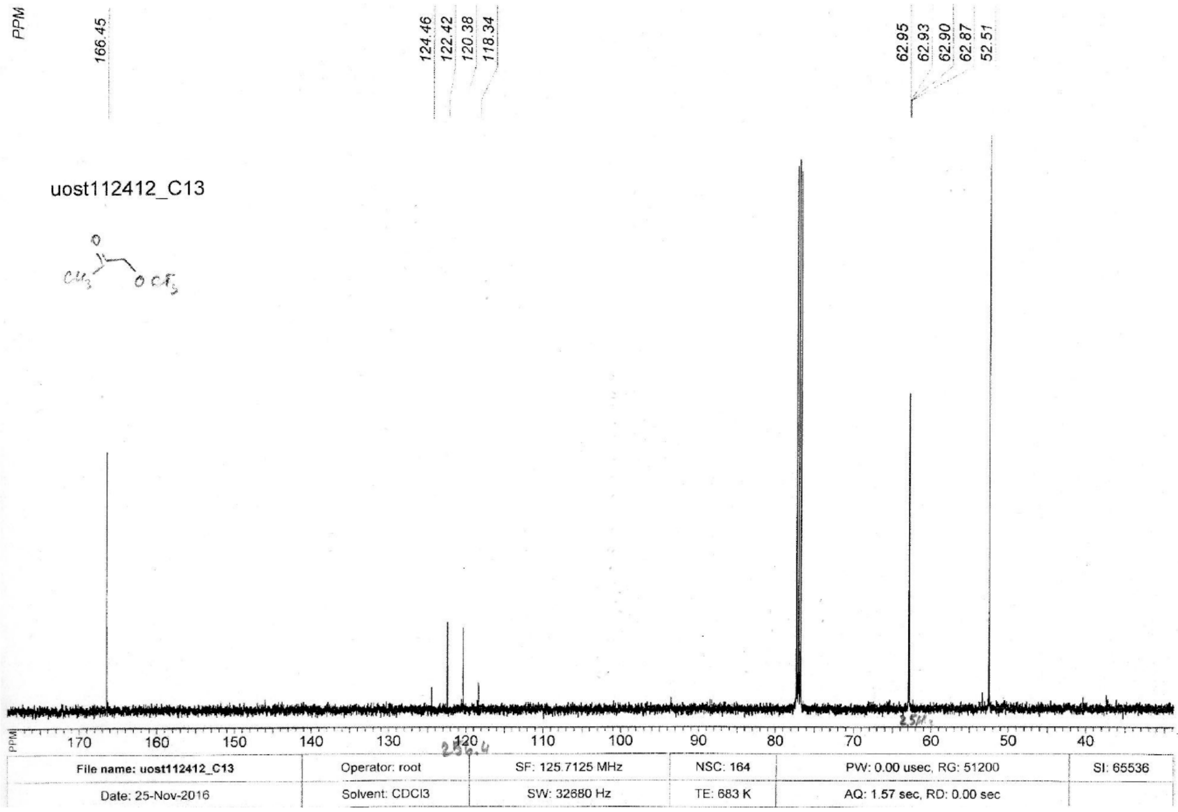

Figure S30. ^{13}C-NMR spectra of methyl (trifluoromethoxy)acetate **3a**.

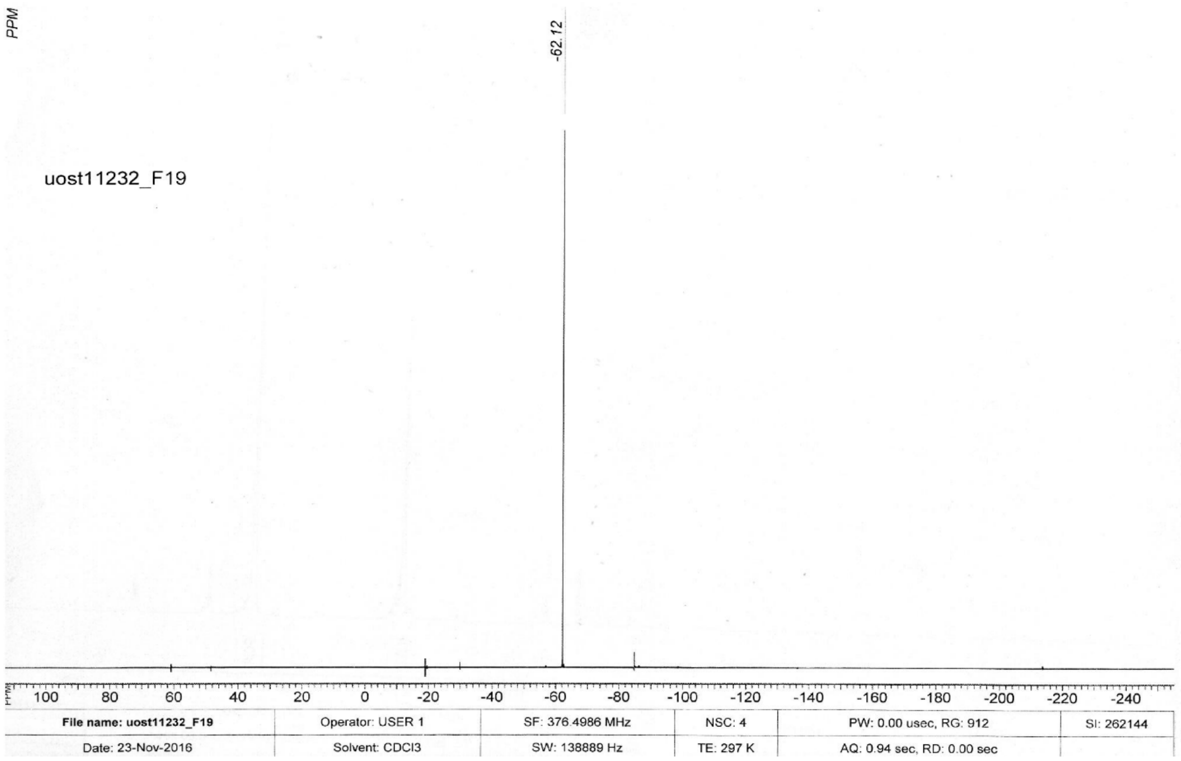

Figure S31. ^{19}F-NMR spectra of methyl (trifluoromethoxy)acetate **3a**.

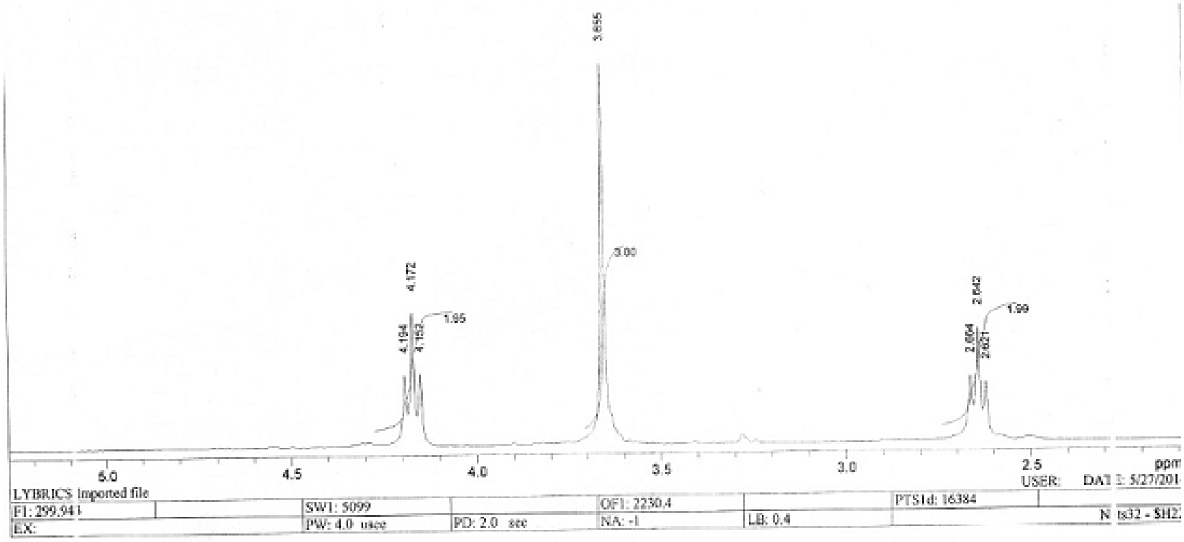

Figure S32. ^{1}H-NMR spectra of methyl 3-(trfluoromethoxy)propanoate **3b**.

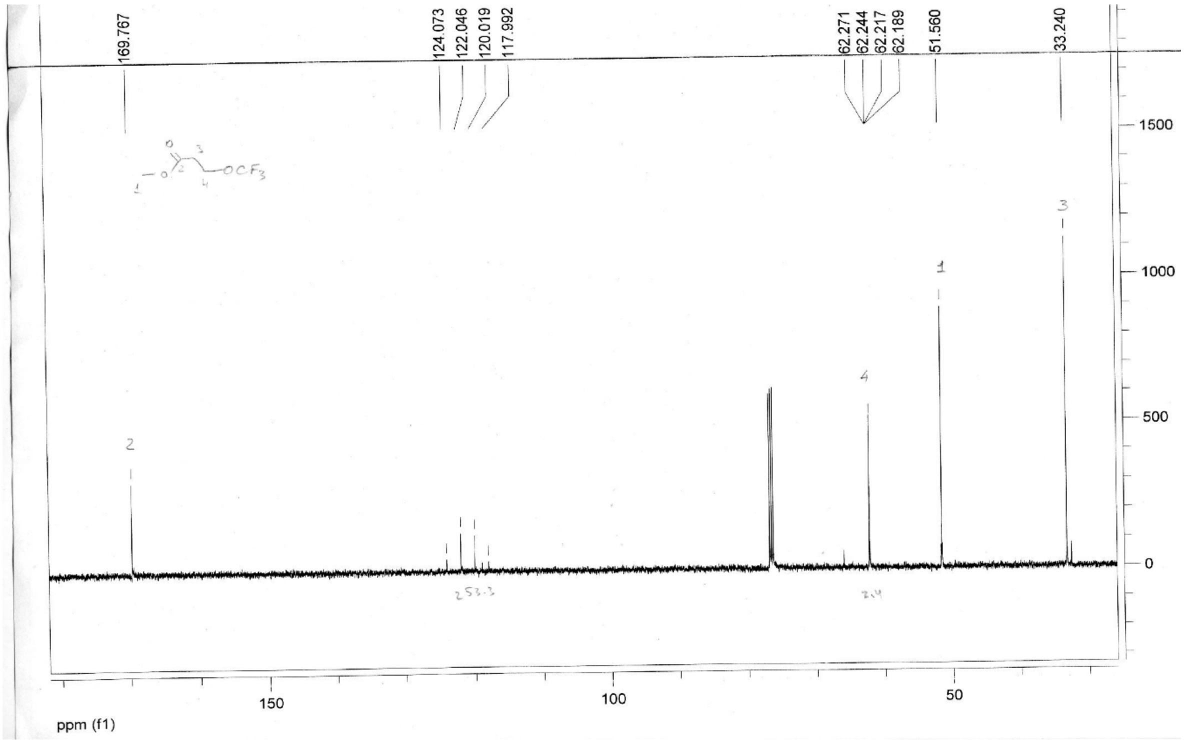

Figure S33. ^{13}C-NMR spectra of methyl 3-(trfluoromethoxy)propanoate **3b**.

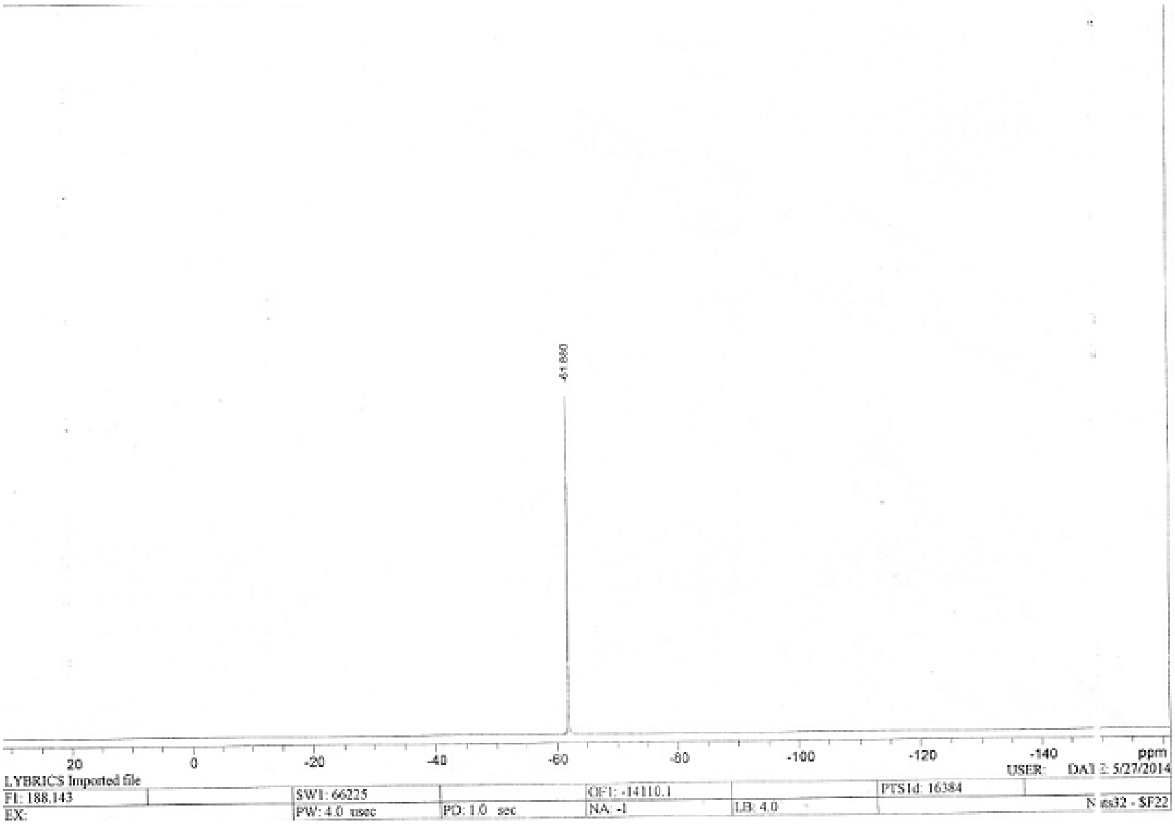

Figure S34. ^{19}F-NMR spectra of methyl 3-(trfluoromethoxy)propanoate **3b**.

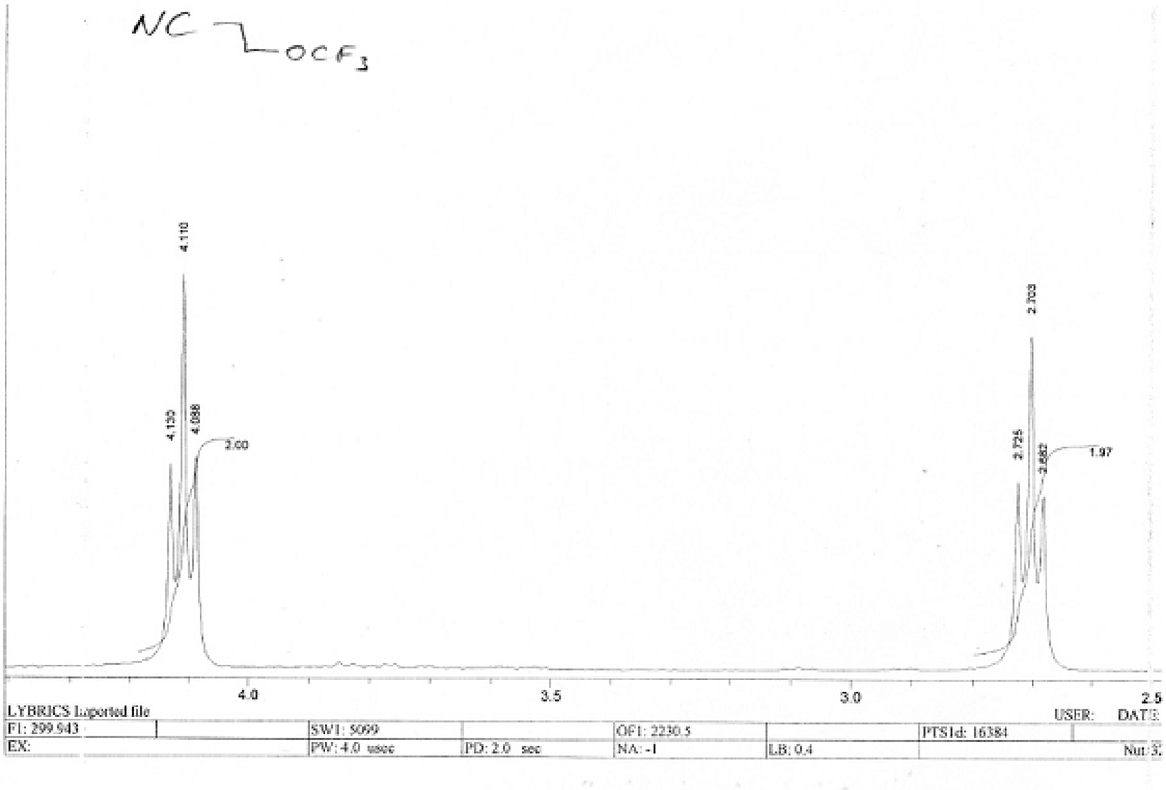

Figure S35. ^{1}H-NMR spectra of 3-(trifluoromethoxy)propanenitrile **3c**.

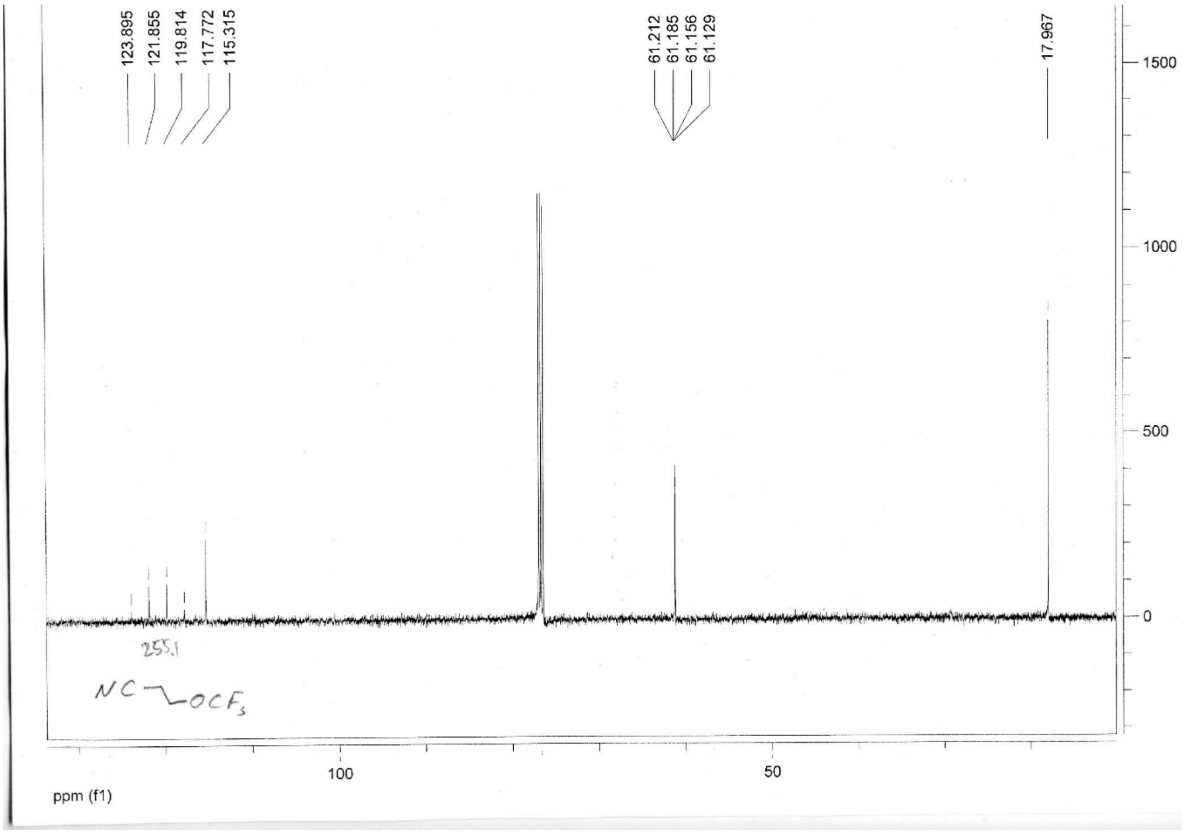

Figure S36. ^{13}C-NMR spectra of 3-(trifluoromethoxy)propanenitrile **3c**.

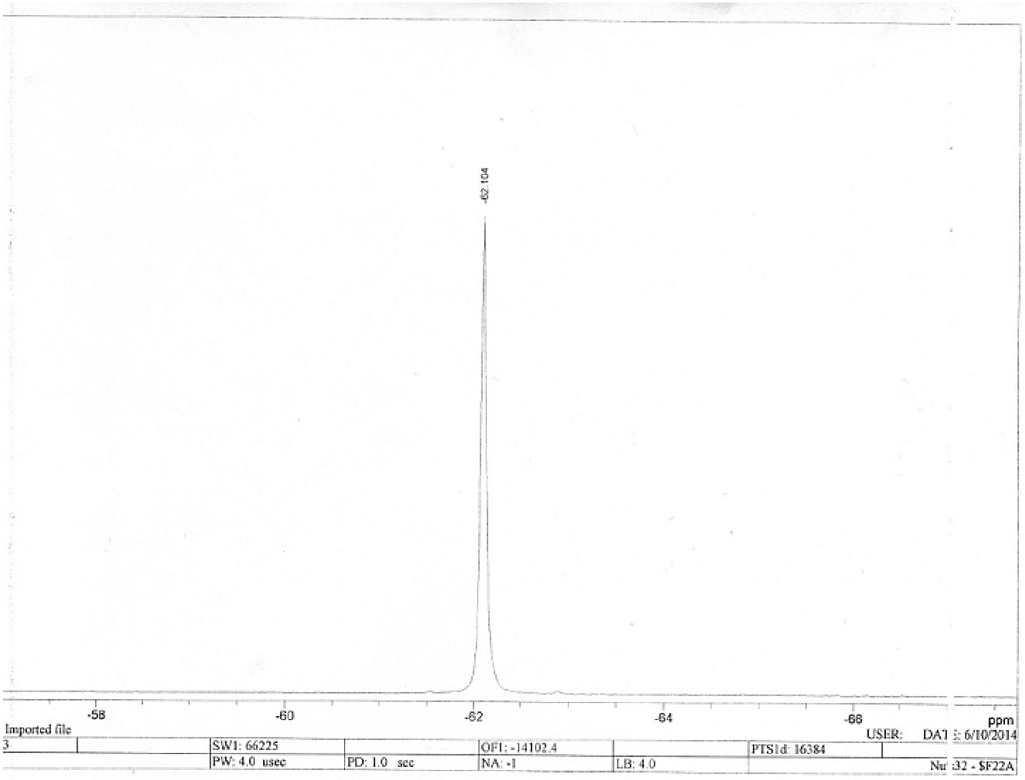

Figure S37. ^{19}F-NMR spectra of 3-(trifluoromethoxy)propanenitrile **3c**.

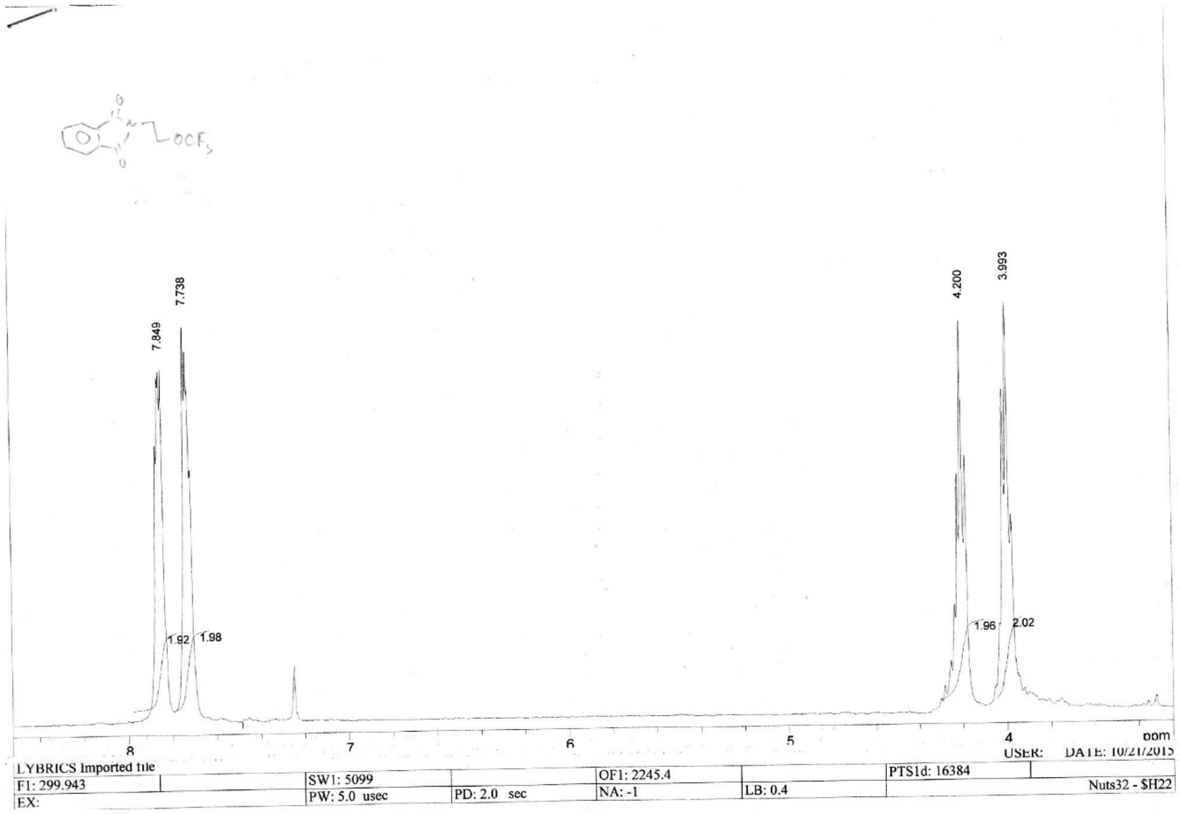

Figure S38. ^{1}H-NMR spectra of 2-[2-(trifluoromethoxy)ethyl]-1H-isoindole-1,3(2H)-dione **3d**.

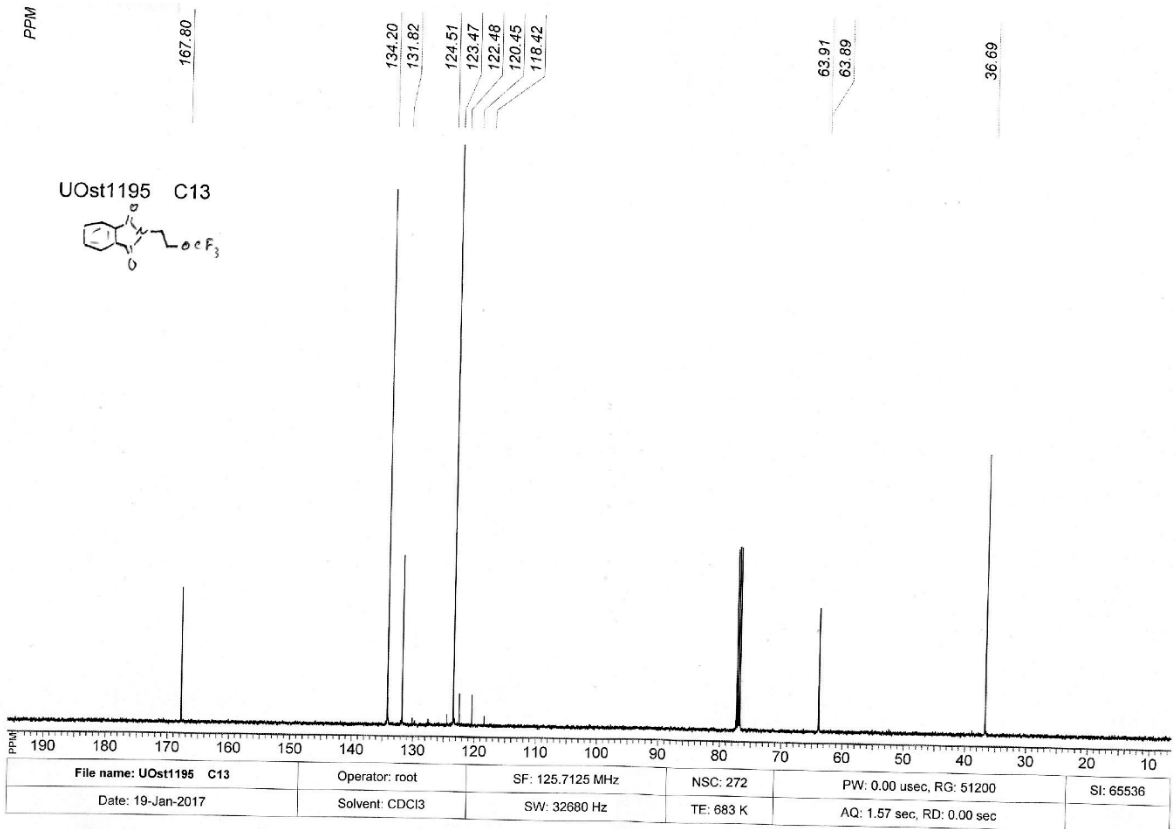

Figure S39. ^{13}C-NMR spectra of 2-[2-(trifluoromethoxy)ethyl]-1H-isoindole-1,3(2H)-dione **3d**.

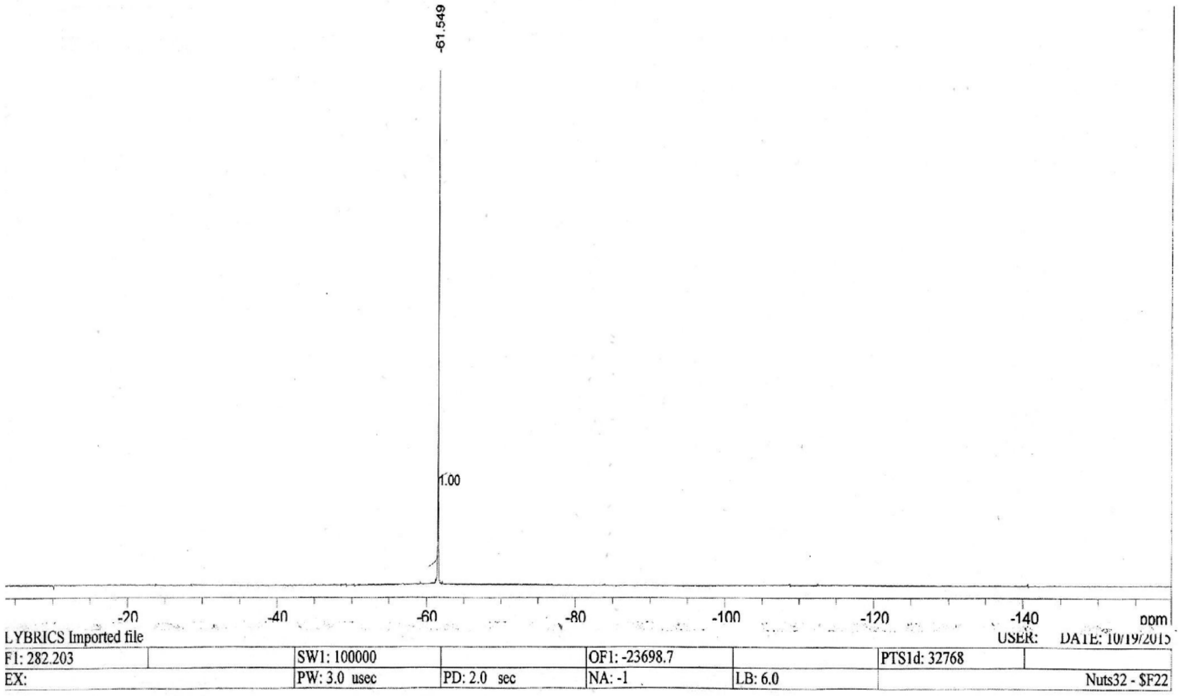

Figure S40. ^{19}F-NMR spectra of 2-[2-(trifluoromethoxy)ethyl]-1H-isoindole-1,3(2H)-dione **3d**.

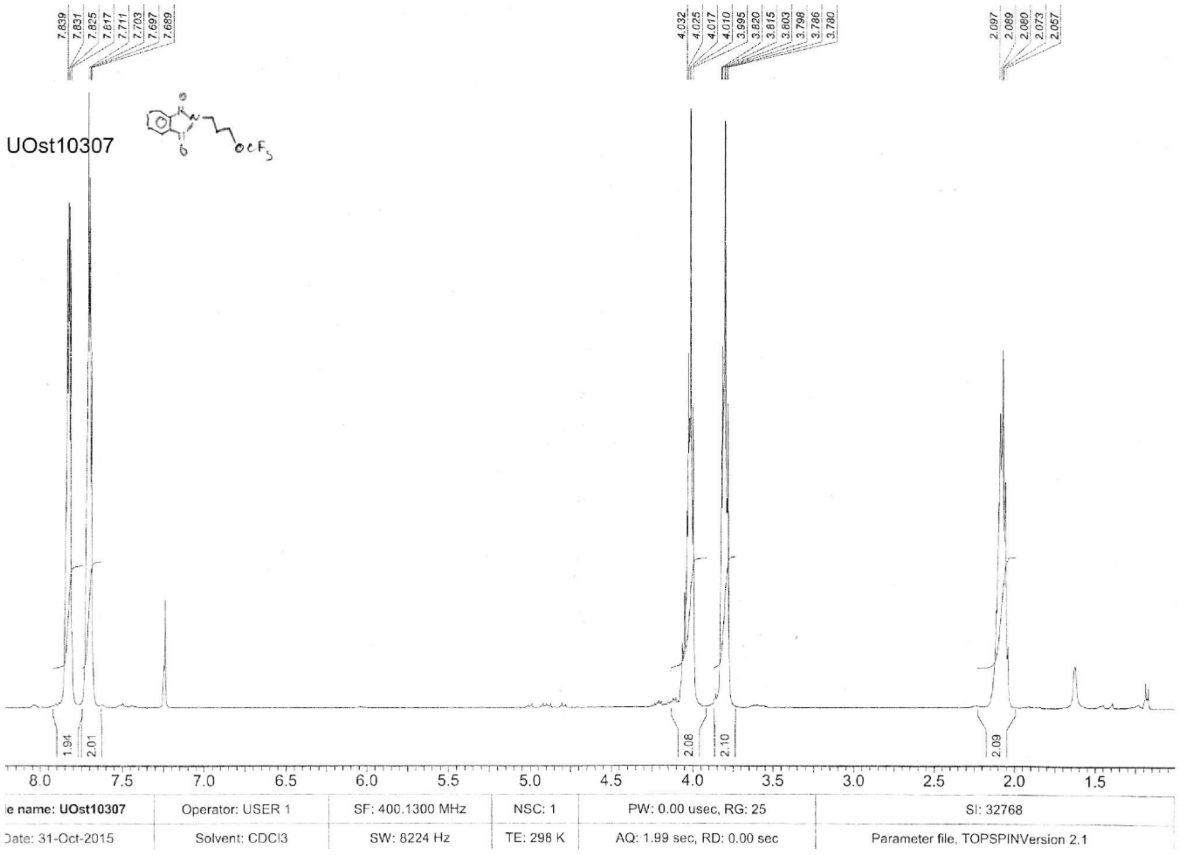

Figure S41. ^{1}H-NMR spectra of 2-[3-(trifluoromethoxy)propyl]-1H-isoindole-1,3(2H)-dione **3e**.

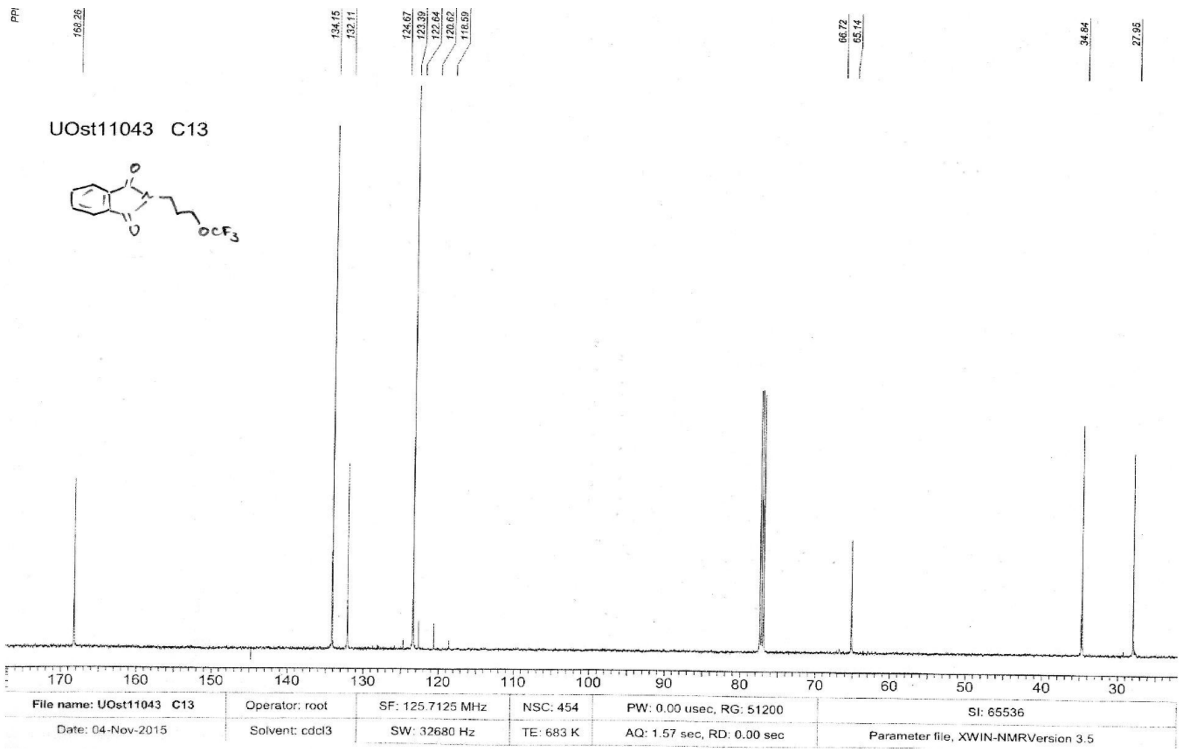

Figure S42. ^{13}C-NMR spectra of 2-[3-(trifluoromethoxy)propyl]-1H-isoindole-1,3(2H)-dione **3e**.

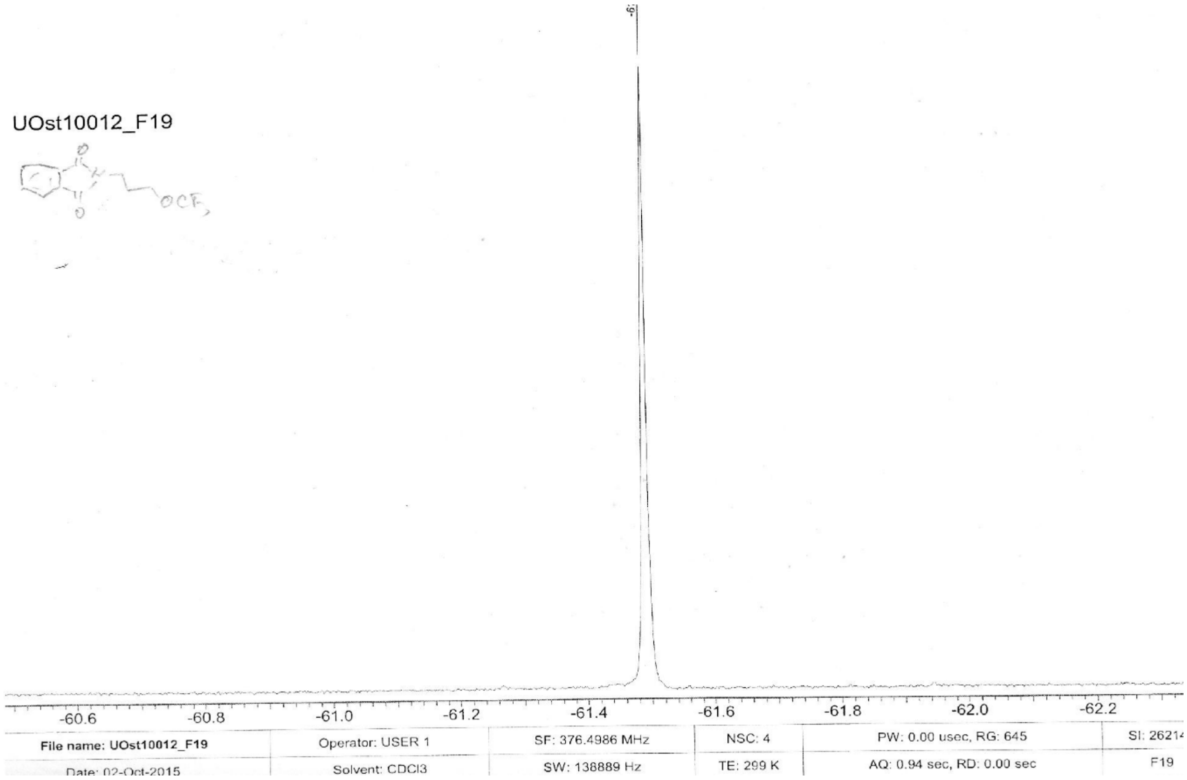

Figure S43. ^{19}F-NMR spectra of 2-[3-(trifluoromethoxy)propyl]-1H-isoindole-1,3(2H)-dione **3e**.

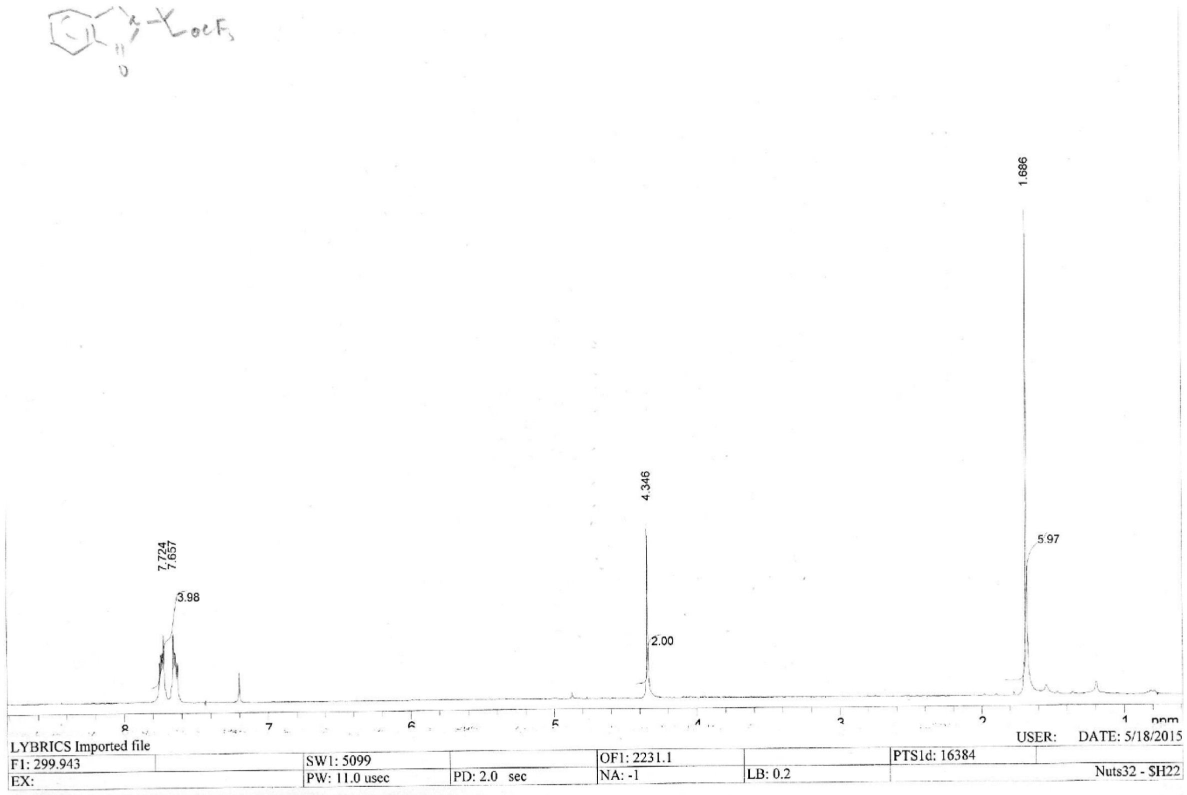

Figure S44. ^{1}H-NMR spectra of 2-[1,1-dimethyl-2-(trifluoromethoxy)ethyl]-1H-isoindole-1,3(2H)-dione **3f**.

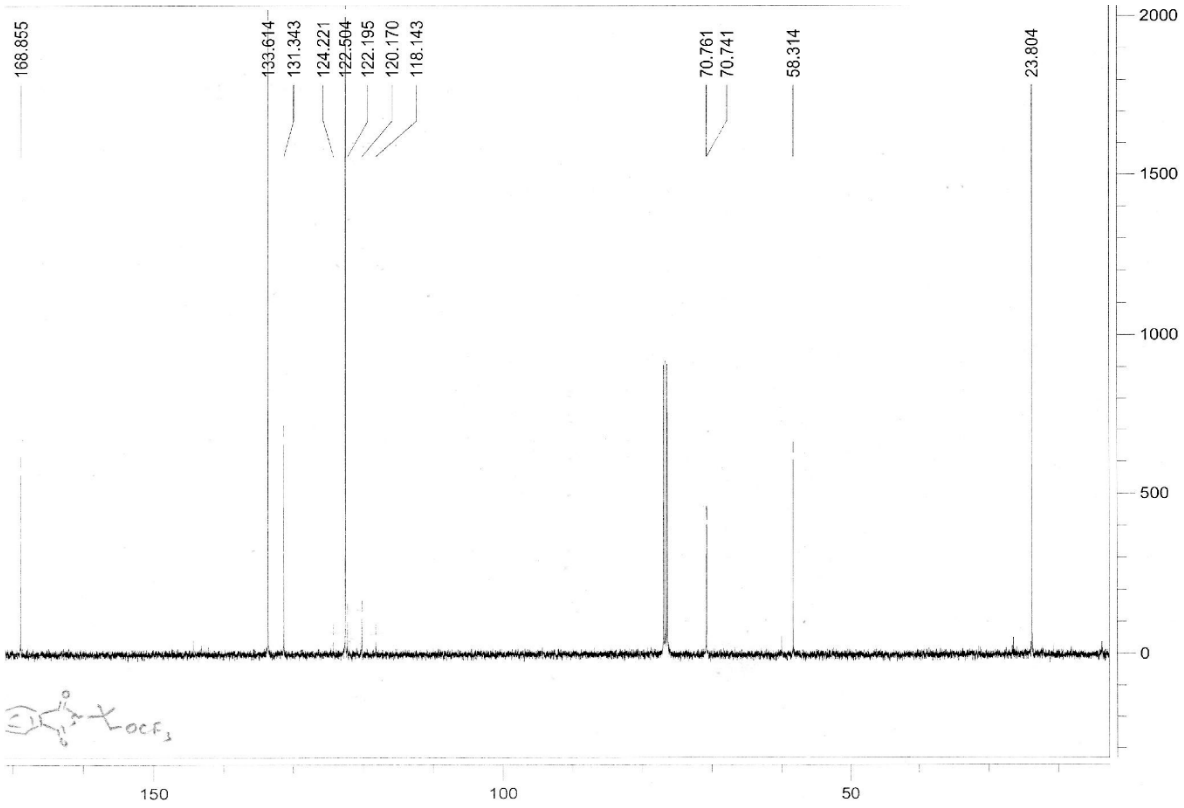

Figure S45. ^{13}C-NMR spectra of 2-[1,1-dimethyl-2-(trifluoromethoxy)ethyl]-1H-isoindole-1,3(2H)-dione **3f**.

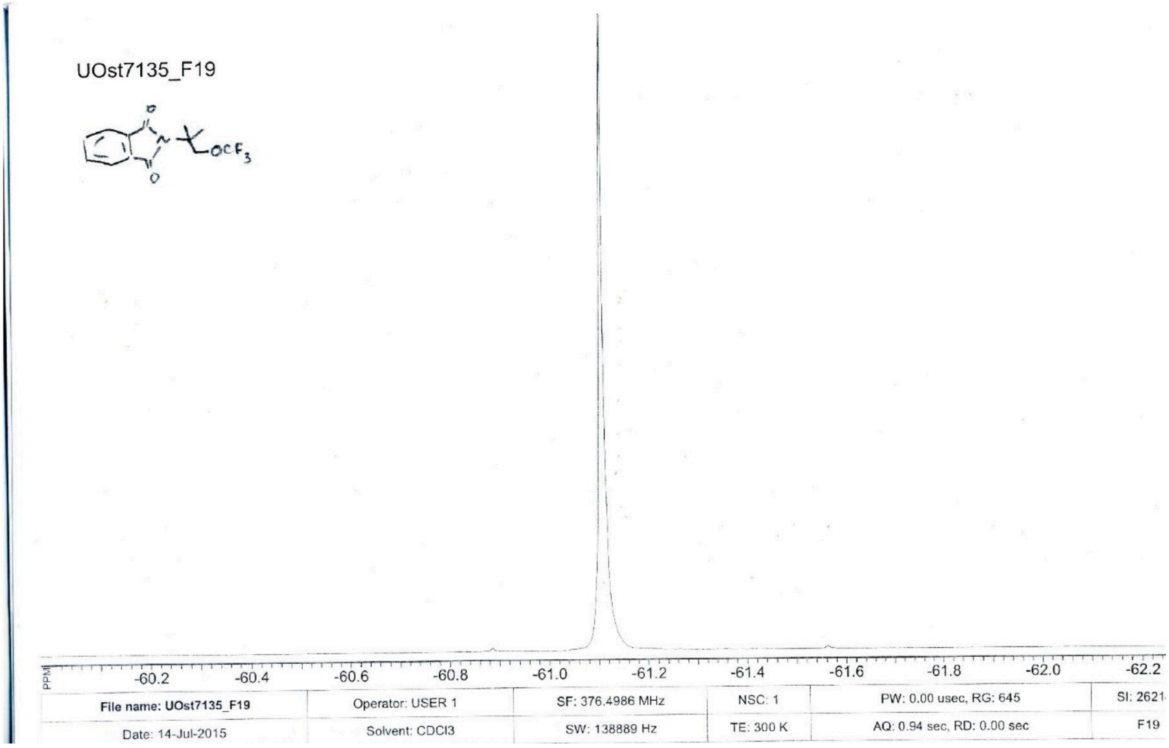

Figure S46. ^{19}F-NMR spectra of 2-[1,1-dimethyl-2-(trifluoromethoxy)ethyl]-1H-isoindole-1,3(2H)-dione **3f**.

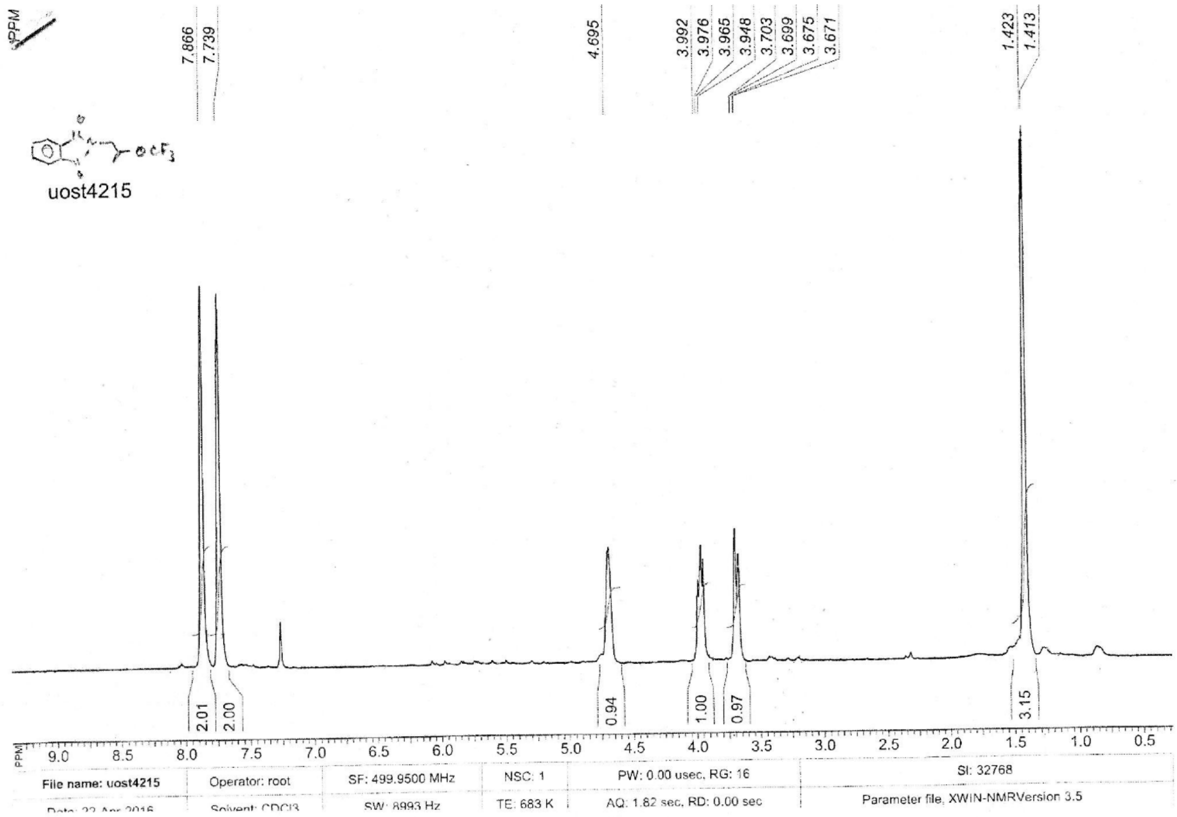

Figure S47. ^{1}H-NMR spectra of 2-[2-(trifluoromethoxy)propyl]-1H-isoindole-1,3(2H)-dione **3g**.

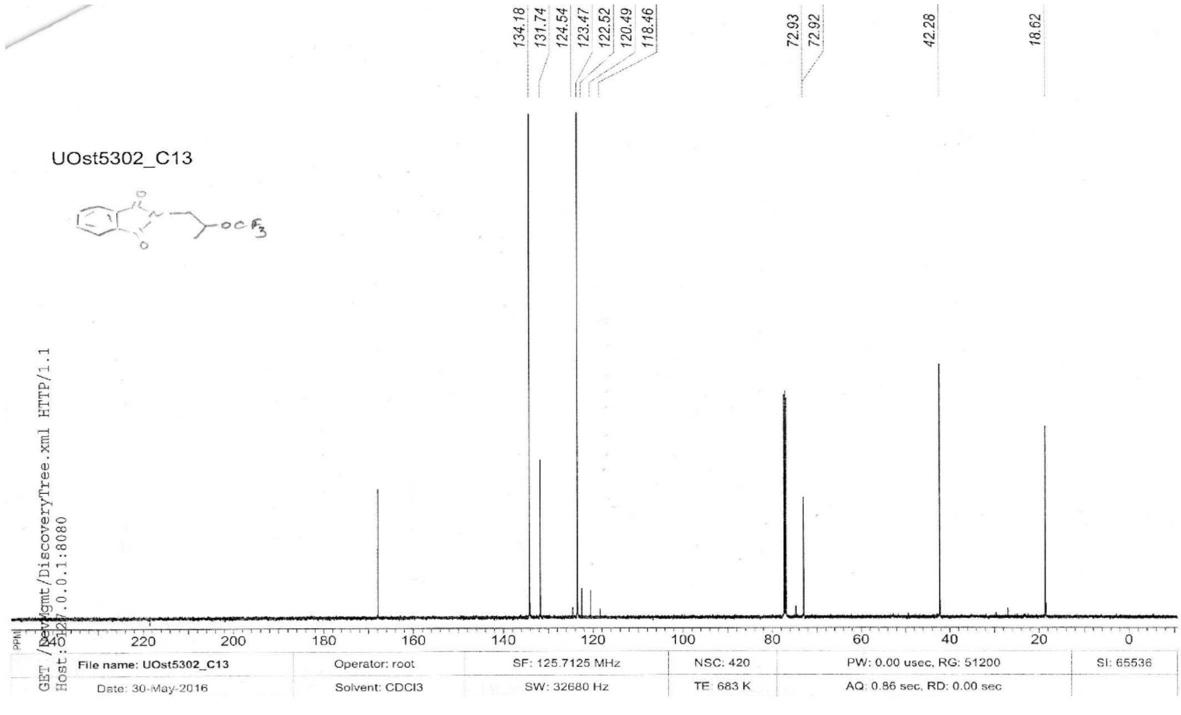

Figure S48. ^{13}C-NMR spectra of 2-[2-(trifluoromethoxy)propyl]-1H-isoindole-1,3(2H)-dione **3g**.

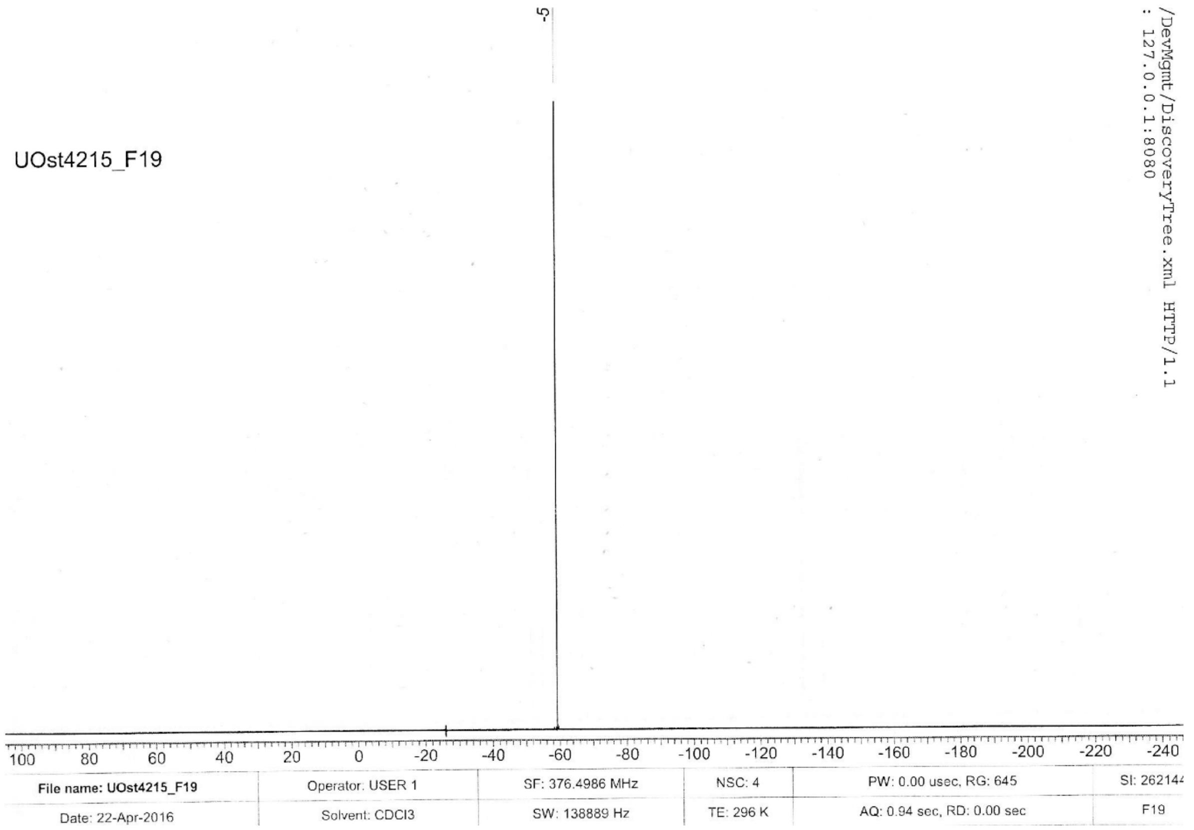

Figure S49. ^{19}F-NMR spectra of 2-[2-(trifluoromethoxy)propyl]-1H-isoindole-1,3(2H)-dione **3g**.
